# Supplementary material for: Roles of BrlA and AbaA in Mediating Asexual and Insect Pathogenic Lifecycles of Metarhizium robertsii
Source: J Fungi (Basel). 2022 Oct 21;8(10):1110. doi: 10.3390/jof8101110 (PMC9604561; doi:10.3390/jof8101110)
Supplement: Supplementary file 1 [file jof-08-01110-s001.zip › jof-1967666-supplementary.pdf]

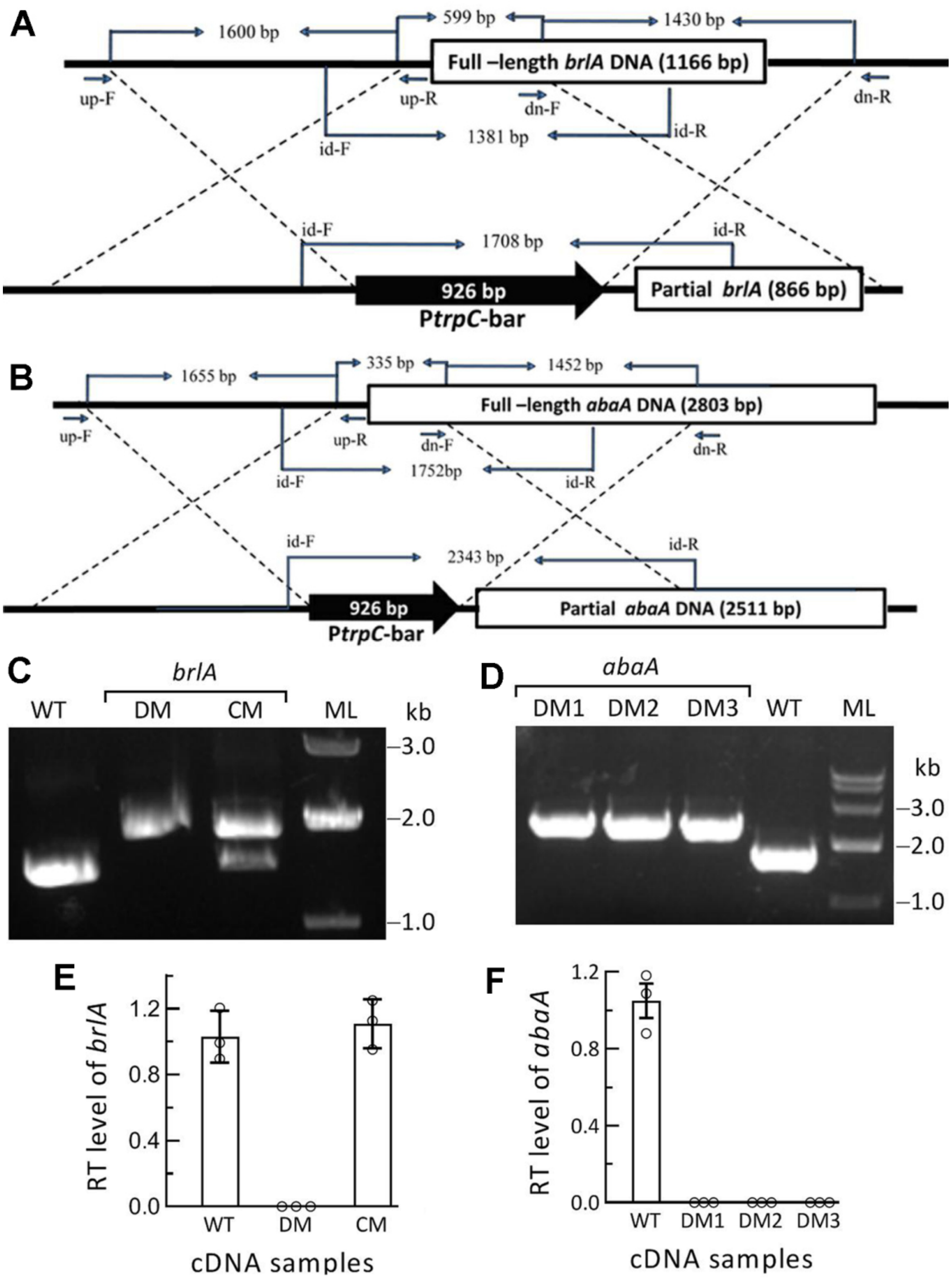

Figure S1. Generation and identification of *brlA* and *abaA mutants* in *M. robertsii.* (**A, B**) Schematic diagrams for the strategies of disruption *brlA* and *abaA* respectively. (**C, D**) The disruption mutants (DM) and complementation mutants (CM) of *brlA* and *abaA* identified via PCR analysis with paired primers (Table S1). The genomic DNA fragments detected by PCR include 2343 and 1753 bp from the *brlA* DM and the wild-type strain (WT) and 2343 and 1752 bp from *abaA* DM1–3 and WT respectively, leading to a partial promoter/coding fragment of *brlA* (1381 + 926 – 1708 = 599 bp) or *abaA* (1752 + 926 – 2343 = 355 bp) deleted from the WT DNA as illustrated in the diagram. (**E, F**) Relative transcript (RT) levels of *brlA* and *abaA* in the cDNA samples of their DM/CM strains with respect to the WT standard. The cDNA samples analyzed via qPCR were derived from three 4-day-old PDA cultures of each strain grown at the optimal regime. Error bars: standard deviations.

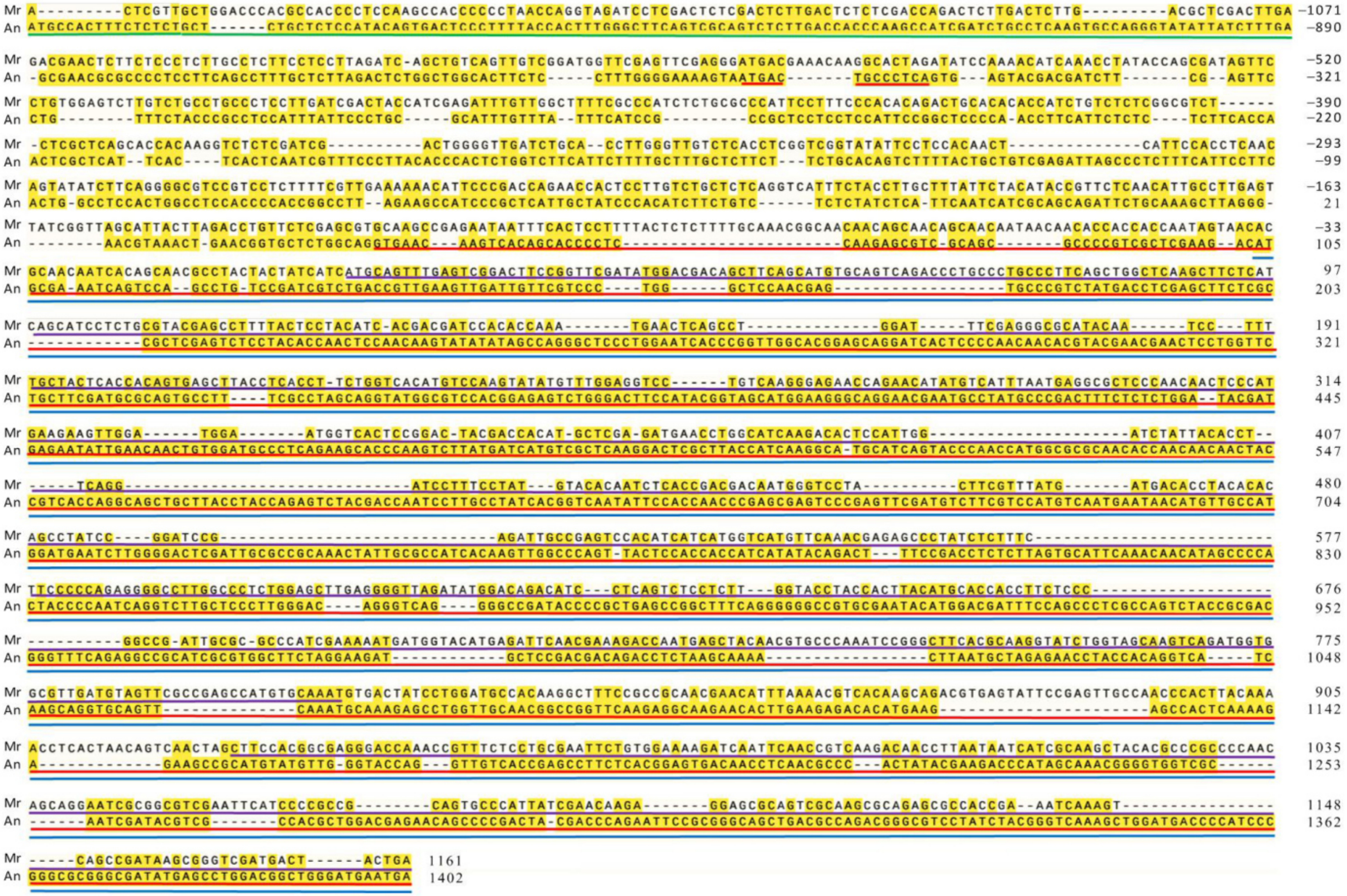

Figure S2. Comparative DNA sequences of three transcription units found in the *brlA* orthologs of *A. nidulans* (An) and *M. robertsii* (Mr). The transcription units *brlA*α, *brlA*β and μORF are underlined regions in blue, red and green respectively. The underlined region in purple represents the coding sequence of *brlA* in *M. robertsii*.

Table S1. Paired primers used for manipulation and detection of *brlA* and *abaA* in *M. robertsii*.

**Primers**	**Paired sequences (5′–3′)***	**Purpose**
cBrlA-F/R	CACAAACACCTTCAAACCCGGGATGCAGTTTGAGTCGGACTTC / TGCTCACCATGTTAACGGATCCGTAGTCATCGACCCGCTTAT	Cloning *brlA* cDNA (1107 bp) for fusion to *gfp*
cAbaA-F/R	CACAAACACCTTCAAACCCGGGATGTCTTCACTCTTTCAG / TGCTCACCATGTTAACGGATCCCCATCCAGCAGTGACGGGCA	Cloning *abaA* cDNA (2658 bp) for fusion to *gfp*
upBrlA-F/R	TAACCCGGGGATCCGCGATGATGTATTGACGGAC / GTACCAAGCTTGGCTGCAGGTGGAATGAGTTGTGGAGGA	Cloning *brlA* 5′-end (1600 bp) for recombination
dnBrlA-F/R	CGTCGACCCATGGCTCGAGCCAACAACTCCCATGAAGAA / CACTAGTCAGATCTTCTAGAGATAATGATGTATGCAGCCAAA	Cloning *brlA* 3′-end (1430 bp) for recombination
upAbaA-F/R	TAACCCGGGGATCCGGTGATAAGGCGGAAGAGG / TGGGAGCTCGGTACCAAGCTTATGAGACGCAACGAACGAG	Cloning *abaA* 5′-end (1655 bp) for recombination
dnAbaA-F/R	CCATGGCTCGAGTCTAGACCAAGACTTCCGTCAATCCC / CGTTAACACTAGTCAGATCTAGCGTCGCCTTCATCCTC	Cloning *abaA* 3′-end (1452 bp) for recombination
flBrlA-F/R	*ATCCGTCGACCTGCAGCCAAGCTT*TCCCTCTTGCCTCTTCCT / *ACACTAGTCAGATCTTCTCTAGA*GAATCCAGCCGCACTATGA	Cloning full-length *brlA* (2105 bp) for rescue
pBrlA-F/R	GAGGGATGACGAAACAAGGC / ATGGCTCGGCGAACTACA	PCR detecting *brlA*
pAbaA-F/R	GGCTTGGTTCTCGTTCGT / GTCGCCATCGGCATACTT	PCR detecting *abaA*
qBrlA-F/R	TCAGCTGGCTCAAGCTTCTC / TCGTAGTCCGGAGTGACCAT	qPCR detecting *brlA*
qAbaA-F/R	GCGTCCTTCCCTCACTTGAA / TAGCAACCTCGACAAACCCC	qPCR detecting *abaA*
18S rRNA-F/R-	GAGCCAGCGAGTAATTCC/ AGCCATTCAATCGGTAGTAG	qPCR detecting 18S rRNA

*Underlined regions denote the restriction enzyme sites introduced for the fusion of *brlA* or *abaA* cDNA (*Xma*I/*Bam*HI) to *gfp* or the deletion of *brlA* or *abaA* via homologous recombination of its *bar*-separated 5′ and 3′ fragments (*Bam*HI/*Pst*I and *Xho*I/*Xma*I). Underlined and italicized regions are the recognition fragments for the gateway exchange in complementary plasmid.

Table S2. A list of differentially expressed genes identified from the Δ*brlA* mutant versus the WT strain of *M. roberstii*.

gene_id	gene_name	FPKM			FPKM			Δ*brlA*/WT			Description
		Δ*brlA*_1	Δ*brlA*_2	Δ*brlA*_3	WT_1	WT_2	WT_3	$\log_2 R$	*p*-value	*q*-value	
19263638	MAA_09352	0.0	0.0	0.0	14.0	30.8	28.1	-17.89	4.07E-17	7.00E-16	hypothetical protein MAA_09352
19263635	MAA_09349	0.0	0.0	0.0	11.7	8.5	8.3	-16.54	1.47E-15	2.21E-14	hypothetical protein X797_010127
19262897	MAA_08611	0.0	0.0	0.0	4.6	5.9	3.7	-15.53	1.89E-08	1.51E-07	hypothetical protein X797_008967
19255917	MAA_01631	0.0	0.0	0.0	1.9	2.7	1.7	-14.38	1.22E-07	8.60E-07	3-octaprenyl-4-hydroxybenzoate carboxy-lyase
19255910	MAA_01624	0.0	0.0	0.0	1.0	2.2	1.8	-14.04	1.95E-06	1.16E-05	LysR family regulatory protein
19262624	MAA_08338	0.0	0.0	0.0	1.8	0.0	1.6	-13.45	5.31E-03	1.55E-02	DUF3712 domain protein
19255911	MAA_01625	0.0	0.0	0.0	1.1	1.3	0.7	-13.34	3.18E-03	9.83E-03	Thioesterase
19264873	MAA_10587	0.0	0.0	0.0	0.0	1.7	1.1	-13.17	3.70E-03	1.12E-02	O-methyltransferase, family 2
23632182	MAA_10733	0.0	0.0	0.0	0.3	0.1	0.7	-11.88	1.55E-03	5.20E-03	FMN-dependent alpha-hydroxy acid dehydrogenase, active site protein
19262594	MAA_08308	0.1	0.0	0.0	17.9	28.0	19.4	-9.84	3.54E-16	5.62E-15	Peptidase aspartic, active site protein
19263637	MAA_09351	137.4	177.8	3.2	48866.7	83933.1	58904.2	-9.23	1.41E-13	1.88E-12	hypothetical protein MAA_09351
19262625	MAA_08339	21.6	29.4	0.0	7189.7	11527.9	8575.0	-9.06	1.82E-07	1.27E-06	hypothetical protein MAA_08339
19259048	MAA_04762	2.3	3.9	1.4	622.7	1036.0	809.9	-8.36	1.07E-67	1.55E-65	Multicopper oxidase, copper-binding site
19259151	MAA_04865	0.6	1.2	0.0	127.5	195.1	109.4	-7.91	8.54E-47	6.67E-45	Peptidoglycan-binding lysin domain protein
19262633	MAA_08347	0.0	0.0	0.0	1.9	1.7	2.7	-7.30	2.15E-04	8.96E-04	phytanoyl-CoA dioxygenase
19255939	MAA_01653	0.0	0.0	0.3	6.4	13.4	23.7	-7.10	4.82E-08	3.60E-07	hypothetical protein MAA_01653
19262656	MAA_08370	0.7	0.0	0.4	23.6	41.8	35.7	-6.64	8.16E-35	4.14E-33	polyketide synthase
19258931	MAA_04645	7.9	3.4	5.7	238.8	548.6	458.8	-6.20	1.51E-35	7.88E-34	GPR1/FUN34/yaaH family protein
19264949	MAA_10665	29.7	22.1	31.7	1578.2	1999.0	1677.9	-5.98	8.00E-305	8.00E-302	hypothetical protein X797_001079
19259162	MAA_04876	0.3	2.7	2.6	91.9	125.6	96.3	-5.80	1.59E-16	2.62E-15	f420-dependent NADP reductase
19262577	MAA_08291	9.3	4.4	17.3	504.1	546.1	421.4	-5.57	1.56E-25	4.81E-24	sulfatase domain-containing protein
19258736	MAA_04450	12.4	9.8	10.9	508.7	612.8	408.8	-5.54	7.49E-183	3.80E-180	Glycoside hydrolase, superfamily
19263636	MAA_09350	0.3	0.1	0.0	6.7	6.7	5.2	-5.52	1.67E-15	2.49E-14	hypothetical protein MAA_09350
19262650	MAA_08364	0.4	0.3	1.2	7.9	27.9	45.9	-5.44	5.87E-12	7.05E-11	IDI-3 protein
19255960	MAA_01674	0.0	0.0	2.2	18.6	42.7	25.5	-5.33	2.38E-03	7.62E-03	Endoribonuclease L-PSP/chorismate mutase-like protein
19264888	MAA_10602	0.8	0.0	0.0	7.1	9.7	11.9	-5.14	3.58E-06	2.02E-05	subtilisin-like protease
19262896	MAA_08610	0.0	0.0	0.3	3.0	4.6	3.2	-5.05	5.49E-07	3.64E-06	Protein kinase domain containing protein
19261999	MAA_07713	0.6	1.1	1.6	20.9	51.3	27.5	-4.93	8.89E-15	1.25E-13	hypothetical protein MAA_07713
19262627	MAA_08341	0.0	0.2	0.5	4.2	11.6	4.6	-4.86	2.73E-08	2.12E-07	hypothetical protein MAA_08341

19262581	MAA_08295	0.0	0.0	0.2	1.2	3.5	0.8	-4.75	3.41E-03	1.04E-02	hypothetical protein MAA_08295
19256645	MAA_02359	178.5	171.2	187.6	4512.0	4602.1	3591.3	-4.56	0.00E+00	0.00E+00	Carbohydrate-binding WSC
19264886	MAA_10600	0.2	0.0	0.3	5.1	5.4	1.5	-4.50	8.95E-06	4.69E-05	proteinrelated to zinc finger protein odd-paired-like (opl)
19256469	MAA_02183	98.0	250.3	596.3	4145.2	9244.2	6771.9	-4.42	4.18E-09	3.66E-08	Glucose-repressible protein Grg1
19255929	MAA_01643	1.8	0.8	2.0	7.4	26.7	61.0	-4.39	1.03E-06	6.38E-06	Cytochrome P450 CYP548V1
19259303	MAA_05017	389.4	229.3	327.9	5408.9	7974.6	6431.8	-4.39	1.87E-103	3.79E-101	Extracellular membrane protein, CFEM domain protein
19263122	MAA_08836	0.6	0.0	0.5	4.8	6.9	9.9	-4.19	2.01E-07	1.38E-06	C6 transcription factor
19262653	MAA_08367	76.6	65.1	47.5	421.8	1297.6	1484.8	-4.08	9.54E-13	1.20E-11	ERG4/ERG24 ergosterol biosynthesis-like protein
19262575	MAA_08289	1.0	0.2	0.0	5.1	4.2	10.6	-4.05	2.89E-05	1.41E-04	cell surface protein (Mas1)
19256722	MAA_02436	56.6	27.8	35.9	262.7	622.4	834.6	-3.84	1.08E-11	1.26E-10	NAD(P)-binding domain protein
19256063	MAA_01777	0.3	0.0	0.0	1.0	1.3	1.7	-3.68	1.13E-02	2.96E-02	GPI anchored dioxygenase
19255928	MAA_01642	0.6	0.9	0.4	2.5	7.9	12.7	-3.63	8.83E-11	9.58E-10	fatty acid synthase subunit alpha reductase
19255925	MAA_01639	1.0	0.9	0.5	3.5	8.0	18.4	-3.63	3.29E-07	2.23E-06	peptide synthetase
19256662	MAA_02376	13.4	13.5	14.2	136.5	202.9	149.7	-3.57	6.85E-77	1.16E-74	Aromatic-ring hydroxylase-like protein
19255931	MAA_01645	13.1	6.8	5.7	31.5	77.6	174.2	-3.47	5.60E-06	3.06E-05	branched-chain-amino-acid aminotransferase TOXF
19262004	MAA_07718	1.9	0.0	1.4	5.5	18.1	12.6	-3.45	7.93E-04	2.90E-03	hypothetical protein MAA_07718
19262572	MAA_08286	1.9	0.5	1.4	8.6	19.1	11.3	-3.37	2.81E-09	2.53E-08	dehydrogenase/reductase SDR family
19262899	MAA_08613	214.2	221.1	217.2	1995.8	2517.0	1934.8	-3.30	3.68E-131	1.07E-128	ntf2-like domain protein
19262028	MAA_07742	0.6	0.0	0.2	2.3	5.5	0.4	-3.30	1.08E-02	2.85E-02	subtilisin-like protease
19256169	MAA_01883	13.0	15.6	11.9	129.0	139.0	128.3	-3.29	9.40E-140	3.18E-137	ABC-2 type transporter
19258918	MAA_04632	35.6	31.0	36.8	260.7	389.9	344.2	-3.27	1.01E-73	1.58E-71	hypothetical protein MAA_04632
19259312	MAA_05026	38.7	41.7	34.4	331.4	404.1	332.5	-3.22	6.62E-102	1.22E-99	galactosyltransferase superfamily protein
19256187	MAA_01901	4.8	6.6	2.6	40.3	50.7	37.7	-3.20	7.93E-18	1.48E-16	DUF967 domain protein
19263688	MAA_09402	0.0	0.3	0.2	0.5	2.3	1.3	-3.12	8.01E-03	2.22E-02	Amino acid/polyamine transporter I
19258932	MAA_04646	19.7	16.0	100.8	227.5	576.8	377.3	-3.11	5.04E-04	1.94E-03	hypothetical protein MAA_04646
19259184	MAA_04898	0.3	0.6	0.3	3.2	3.0	3.9	-3.11	3.73E-14	5.09E-13	ABC efflux transporter
19258976	MAA_04690	1.7	3.9	2.5	15.6	24.0	29.7	-3.10	6.19E-16	9.66E-15	DUF1479 domain protein
19255923	MAA_01637	2.0	3.4	0.9	5.8	18.9	28.4	-3.07	7.31E-06	3.89E-05	hypothetical protein MAA_01637
19262576	MAA_08290	0.6	1.1	3.7	9.2	19.1	15.2	-3.02	6.17E-06	3.34E-05	hypothetical protein MAA_08290
19264986	MAA_10702	0.7	2.1	2.0	12.2	11.5	11.2	-2.88	1.69E-11	1.94E-10	D-xylulose reductase A
19264885	MAA_10599	172.4	117.7	193.3	1348.0	1116.6	1019.5	-2.85	5.15E-56	5.22E-54	leucine rich repeat domain containing protein
19255924	MAA_01638	1.0	4.3	1.1	6.1	12.5	27.5	-2.84	1.52E-03	5.13E-03	canalicular multispecific organic anion transporter 1
19256017	MAA_01731	185.4	121.8	174.8	1123.5	1211.9	964.7	-2.78	4.73E-61	5.33E-59	multicopper oxidase

23632196	MAA_10747	22.4	33.1	28.9	76.4	216.1	266.3	-2.73	5.98E-07	3.91E-06	ankyrin repeat protein
19264926	MAA_10641	95.7	96.7	91.3	630.6	652.9	577.2	-2.71	2.62E-123	6.64E-121	carbohydrate esterase family 1 protein
19259015	MAA_04729	50.9	52.7	57.2	179.2	410.9	454.8	-2.70	9.32E-11	1.01E-09	duf500 domain containing protein
19261958	MAA_07672	0.0	1.9	2.4	6.9	8.9	10.9	-2.64	1.21E-02	3.15E-02	hypothetical protein MAA_07672
19264993	MAA_10709	7.6	7.9	5.1	43.2	45.7	38.1	-2.63	1.21E-33	5.99E-32	nicotinamide N-methyltransferase
19256325	MAA_02039	41.9	42.2	43.0	239.7	298.2	228.3	-2.59	1.73E-61	2.07E-59	Glutathione S-transferase/chloride channel
19262652	MAA_08366	83.3	143.3	65.1	247.8	656.8	785.5	-2.53	6.33E-06	3.42E-05	methyltransferase
19262027	MAA_07741	363.4	302.1	223.8	1583.4	1903.4	1664.4	-2.53	7.40E-45	5.36E-43	Formate/nitrite transporter
23632178	MAA_10729	5.7	5.2	4.4	25.5	36.7	20.1	-2.43	2.62E-21	6.18E-20	hypothetical protein MAA_10729
19256738	MAA_02452	1.8	2.0	0.6	6.2	7.7	8.7	-2.37	3.30E-04	1.33E-03	alcohol dehydrogenase
19262905	MAA_08619	16.2	16.8	10.8	70.6	81.6	65.8	-2.32	4.72E-27	1.60E-25	choline transport protein
19256654	MAA_02368	15.6	20.5	34.6	57.2	164.2	126.5	-2.30	2.06E-07	1.42E-06	hypothetical protein MAA_02368
19262569	MAA_08283	0.2	0.2	0.2	0.5	0.7	1.6	-2.28	3.07E-04	1.25E-03	Beta-ketoacyl synthase
19255915	MAA_01629	3.4	1.4	5.9	12.8	15.1	21.3	-2.20	4.85E-05	2.25E-04	GCN5-related N-acetyltransferase (GNAT) domain protein
19263676	MAA_09390	163.2	139.5	159.6	709.1	784.2	611.8	-2.19	2.69E-51	2.48E-49	putative protein family Cys-rich
19259267	MAA_04981	7.2	7.5	4.4	29.1	31.4	25.2	-2.16	9.05E-15	1.27E-13	Extracellular membrane protein, 8-cysteine region, CFEM
19259195	MAA_04909	32.1	40.7	36.3	133.5	185.0	167.0	-2.15	1.12E-35	6.16E-34	major facilitator superfamily transporter
19255927	MAA_01641	9.4	15.7	7.6	30.6	41.4	71.8	-2.14	1.53E-08	1.26E-07	ankyrin repeat protein
19263091	MAA_08805	5.3	4.9	7.7	22.9	33.2	22.1	-2.12	8.93E-07	5.66E-06	IDI-2 precursor
19256376	MAA_02090	69.2	55.2	68.1	236.9	338.9	261.1	-2.12	1.91E-29	7.58E-28	GCN5-related N-acetyltransferase (GNAT) domain protein
19256313	MAA_02027	5.6	5.4	4.2	15.0	26.4	22.2	-2.07	3.84E-09	3.40E-08	hypothetical protein MAA_02027
19259175	MAA_04889	67.2	41.9	58.8	204.1	284.2	215.1	-2.07	3.00E-23	8.23E-22	salicylate hydroxylase
19258785	MAA_04499	87.8	74.5	103.4	299.6	441.2	362.5	-2.05	1.60E-30	7.05E-29	pyruvate decarboxylase
19256326	MAA_02040	149.9	196.0	189.6	584.6	723.2	902.8	-2.05	7.51E-31	3.39E-29	hypothetical protein MAA_02040
19256699	MAA_02413	154.4	105.8	101.0	551.1	424.9	499.3	-2.03	3.08E-29	1.16E-27	C4-dicarboxylate transporter/malic acid transporter
19256739	MAA_02453	25.1	21.0	21.2	76.6	114.5	83.3	-2.03	1.36E-26	4.38E-25	arrestin domain-containing protein
19262613	MAA_08327	100.2	109.6	74.7	329.4	464.6	352.9	-2.01	7.60E-25	2.20E-23	hypothetical protein MAA_08327
19262628	MAA_08342	2.7	1.1	0.6	4.4	7.9	5.1	-1.98	9.83E-04	3.54E-03	Heterokaryon incompatibility
19259129	MAA_04843	22.2	9.4	29.6	74.9	94.0	71.8	-1.97	4.38E-09	3.80E-08	Basic-leucine zipper (bZIP) transcription factor
19259294	MAA_05008	4.8	3.9	4.4	16.3	18.1	16.7	-1.97	1.96E-13	2.55E-12	taurine catabolism dioxygenase TauD
19258936	MAA_04650	27.2	23.9	37.3	133.9	114.6	95.8	-1.96	1.64E-18	3.19E-17	hypothetical protein MAA_04650
19258750	MAA_04464	32.1	32.7	35.0	97.7	148.5	139.9	-1.95	4.06E-26	1.27E-24	Ytp1 and DUF2427 domain protein
19256403	MAA_02117	122.6	124.4	149.5	531.4	454.0	459.4	-1.87	8.45E-59	9.03E-57	Short-chain dehydrogenase/reductase SDR

19262856	MAA_08570	86.1	66.7	87.8	225.4	374.2	270.5	-1.85	2.00E-16	3.25E-15	glyoxalase family protein
19262578	MAA_08292	33.8	37.1	29.5	113.8	134.5	113.1	-1.85	2.80E-29	1.07E-27	Nitrilase/cyanide hydratase and apolipoprotein N-acyltransferase
19259203	MAA_04917	28.3	19.4	28.7	85.2	101.1	71.2	-1.75	8.44E-10	8.16E-09	Stress responsive alpha-beta barrel
19258996	MAA_04710	91.3	60.5	86.7	171.3	319.4	312.5	-1.75	3.76E-11	4.23E-10	Glutathione S-transferase/chloride channel
19262014	MAA_07728	71.3	84.9	60.6	227.0	266.7	236.2	-1.75	1.99E-28	7.20E-27	transmembrane amino acid transporter
19262022	MAA_07736	28.2	22.2	22.7	84.7	62.5	97.1	-1.74	2.68E-23	7.44E-22	Carbohydrate-binding WSC
19263127	MAA_08841	5.0	2.5	2.5	11.9	12.2	9.1	-1.74	4.13E-06	2.30E-05	NAD(P)-binding domain protein
19261930	MAA_07644	146.7	154.2	144.0	423.1	540.6	513.6	-1.73	8.17E-38	5.53E-36	hypothetical protein MAA_07644
19259152	MAA_04866	48.8	31.8	39.7	120.5	148.7	129.0	-1.73	8.38E-18	1.55E-16	SAM-dependent methyltransferase
19256253	MAA_01967	11.4	15.4	8.4	41.2	34.7	37.9	-1.69	2.08E-10	2.15E-09	hypothetical protein MAA_01967
19256318	MAA_02032	16.0	15.3	16.7	47.6	63.1	43.9	-1.69	7.64E-17	1.28E-15	3-phytase
19258823	MAA_04537	19.7	16.9	26.3	65.8	74.1	61.5	-1.68	5.59E-18	1.07E-16	Major facilitator superfamily domain protein
19258830	MAA_04544	42.3	28.4	32.1	51.0	111.2	165.7	-1.67	2.52E-05	1.24E-04	hypothetical protein MAA_04544
19258800	MAA_04514	65.4	86.4	58.2	211.4	227.1	231.3	-1.67	1.97E-25	5.97E-24	metallopeptidase
19264922	MAA_10637	74.9	74.5	72.7	183.0	273.3	251.2	-1.67	1.73E-22	4.44E-21	hypothetical protein MAA_10637
19262657	MAA_08371	56.8	39.7	47.8	90.1	167.4	191.1	-1.64	1.83E-09	1.70E-08	oxidoreductase
19262907	MAA_08621	2.2	1.9	1.3	6.1	3.9	6.9	-1.62	2.62E-03	8.23E-03	f420-dependent NADP reductase
19256766	MAA_02480	30.6	40.2	35.4	104.8	120.2	96.9	-1.60	1.66E-26	5.28E-25	NAD-specific glutamate dehydrogenase
23632203	MAA_10754	7.4	5.2	5.4	19.6	13.0	21.5	-1.59	1.01E-06	6.31E-06	gag-pol polyprotein
19259036	MAA_04750	18.5	14.6	15.3	36.6	55.4	52.9	-1.58	7.00E-09	5.99E-08	
19263677	MAA_09391	14.5	13.4	13.0	36.3	45.2	39.2	-1.56	2.35E-27	8.35E-26	hypothetical protein MAA_09391
19259268	MAA_04982	18.5	9.4	7.6	32.8	35.1	36.9	-1.56	1.82E-06	1.10E-05	protein-arginine deiminase type-4
19264893	MAA_10607	3.3	3.6	5.8	10.4	13.2	13.7	-1.56	6.84E-06	3.66E-05	hypothetical protein MAA_10607
19258999	MAA_04713	26.6	28.2	37.8	60.1	116.4	95.0	-1.55	3.18E-07	2.16E-06	hypothetical protein MAA_04713
19259273	MAA_04987	20.3	15.9	13.9	43.7	62.1	40.9	-1.55	2.03E-06	1.21E-05	Kazal domain-containing protein
19259271	MAA_04985	39.8	39.0	32.6	73.3	140.3	112.3	-1.55	1.55E-08	1.28E-07	NADPH-dependent FMN reductase
19261986	MAA_07700	1.8	1.2	2.2	4.4	3.8	7.2	-1.54	1.67E-03	5.54E-03	FAD-binding protein
19259159	MAA_04873	3.0	5.4	4.3	12.5	13.3	11.0	-1.54	1.88E-06	1.13E-05	acetolactate synthase
19264975	MAA_10691	7.2	3.4	6.8	15.0	18.6	16.8	-1.53	1.38E-05	7.03E-05	S-adenosyl-L-methionine-dependent methyltransferase
19262909	MAA_08623	43.1	46.6	48.6	165.8	106.4	127.7	-1.53	4.00E-17	6.93E-16	phytanoyl-CoA dioxygenase
19259160	MAA_04874	58.5	39.2	51.3	157.5	138.2	130.9	-1.52	9.59E-19	1.91E-17	glycosyltransferase family 31
19262021	MAA_07735	18.8	19.0	19.0	53.4	47.4	58.6	-1.49	6.64E-24	1.87E-22	Extracellular membrane protein, CFEM domain protein
19256319	MAA_02033	5.8	5.7	14.1	27.1	28.5	16.0	-1.49	7.80E-03	2.16E-02	hypothetical protein MAA_02033

19262026	MAA_07740	12.0	5.5	13.1	23.9	36.5	24.8	-1.48	5.25E-05	2.43E-04	muconate cycloisomerase I, MLE
19256283	MAA_01997	24.1	27.6	21.8	64.9	74.5	62.6	-1.46	5.22E-17	8.83E-16	Carboxylesterase type B, active site protein
19259247	MAA_04961	13.8	10.1	21.5	43.8	46.0	34.6	-1.45	7.13E-07	4.56E-06	vacuolar triacylglycerol lipase
19256663	MAA_02377	16.9	36.5	20.3	61.8	53.3	85.8	-1.45	7.29E-04	2.70E-03	ATP-binding cassette transporter ifT1
19256755	MAA_02469	123.3	118.7	138.9	335.6	376.0	323.3	-1.44	9.78E-37	6.02E-35	acetoacetyl-CoA synthase
19264976	MAA_10692	11.0	3.7	4.1	17.0	17.3	15.9	-1.42	9.64E-03	2.59E-02	ubiquitin-conjugating enzyme
19259171	MAA_04885	74.5	81.0	55.0	142.8	221.8	196.5	-1.41	3.68E-10	3.70E-09	Short-chain dehydrogenase/reductase SDR
19256224	MAA_01938	21.3	12.1	13.1	37.5	46.9	39.4	-1.41	7.30E-07	4.65E-06	thioesterase family protein
19263633	MAA_09347	7.3	4.6	8.4	15.8	22.7	15.2	-1.40	1.55E-04	6.71E-04	transmembrane amino acid transporter
19261985	MAA_07699	8.6	9.0	8.4	16.5	23.2	29.2	-1.40	1.28E-07	9.05E-07	flavin-binding monooxygenase
19256457	MAA_02171	142.2	188.6	147.3	397.9	435.6	397.8	-1.37	1.96E-22	4.97E-21	Calcium/proton exchanger
19262850	MAA_08564	41.6	41.2	43.1	113.0	106.8	101.9	-1.35	7.22E-48	6.11E-46	IQ calmodulin-binding motif domain protein
19264954	MAA_10670	34.7	38.2	37.0	53.2	96.5	130.2	-1.35	4.43E-05	2.08E-04	hypothetical protein MAA_10670
19262626	MAA_08340	24.2	25.1	22.4	52.9	70.7	57.6	-1.34	5.31E-15	7.75E-14	hypothetical protein MAA_08340
19264965	MAA_10681	6.5	7.0	2.5	8.7	17.8	14.1	-1.34	6.28E-03	1.79E-02	hypothetical protein MAA_10681
19264966	MAA_10682	14.6	18.4	18.8	35.5	42.6	52.7	-1.34	1.34E-11	1.54E-10	WD40 domain protein
23632179	MAA_10730	44.3	37.5	40.7	82.4	129.8	93.5	-1.32	1.05E-08	8.81E-08	Bromodomain protein
19256239	MAA_01953	3.2	6.7	2.5	9.7	10.0	11.2	-1.32	3.41E-03	1.04E-02	dipeptidyl aminopeptidase/acylaminoacyl peptidase
19258977	MAA_04691	172.3	115.6	163.1	227.8	446.9	447.3	-1.31	1.73E-06	1.05E-05	hypothetical protein H634G_00721
19256669	MAA_02383	91.8	93.6	86.2	204.3	228.4	241.1	-1.31	6.04E-30	2.61E-28	homogentisate 1,2-dioxygenase
19263682	MAA_09396	326.5	329.9	375.0	763.4	971.8	804.9	-1.30	8.91E-23	2.38E-21	Alcohol dehydrogenase superfamily, zinc-type
19258909	MAA_04623	38.4	48.8	34.9	82.5	105.5	112.5	-1.30	1.74E-11	1.98E-10	glutamate decarboxylase
19256221	MAA_01935	28.3	18.8	23.5	44.4	58.0	70.6	-1.29	1.63E-08	1.33E-07	integral membrane protein
19256496	MAA_02210	170.1	139.5	141.7	337.4	400.9	360.6	-1.28	1.31E-20	2.96E-19	glucosamine-6-phosphate deaminase
19259074	MAA_04788	63.7	49.3	71.0	113.4	178.6	154.2	-1.28	2.99E-08	2.31E-07	dopa 4,5-dioxygenase
19263092	MAA_08806	10.9	11.9	16.5	34.4	39.2	21.4	-1.27	4.05E-05	1.93E-04	Aminoglycoside phosphotransferase
19263651	MAA_09365	152.2	130.0	129.8	236.2	424.8	331.9	-1.27	3.88E-09	3.42E-08	NAD dependent epimerase/dehydratase family protein
19264994	MAA_10710	6.3	7.7	4.1	10.8	18.6	14.3	-1.27	1.93E-03	6.36E-03	hypothetical protein MAA_10710
19263667	MAA_09381	34.0	34.2	10.2	43.7	76.0	69.0	-1.27	5.08E-03	1.50E-02	protoporphyrinogen oxidase
19259052	MAA_04766	218.4	199.2	162.9	477.6	472.4	446.3	-1.27	2.37E-20	5.22E-19	4-hydroxyphenylpyruvate dioxygenase
19264931	MAA_10646	50.1	42.6	45.3	112.3	100.0	119.5	-1.27	1.54E-21	3.68E-20	hypothetical protein MAA_10646
19263093	MAA_08807	9.1	5.9	12.1	27.5	21.5	16.0	-1.26	2.03E-04	8.52E-04	Alkaline phosphatase, active site protein
23632200	MAA_10751	23.2	18.3	27.6	71.8	46.9	46.1	-1.25	1.13E-05	5.85E-05	

19255941	MAA_01655	31.3	31.3	15.6	33.6	74.6	76.9	-1.24	7.76E-04	2.85E-03	hypothetical protein MAA_01655
19262655	MAA_08369	608.2	573.6	706.5	637.9	1816.1	1993.5	-1.24	1.68E-02	4.18E-02	Dimeric alpha-beta barrel
19261929	MAA_07643	19.0	16.4	22.0	46.8	45.7	42.2	-1.23	2.85E-12	3.46E-11	alternative sulfate transporter
19261964	MAA_07678	15.1	9.8	15.0	27.7	28.7	36.5	-1.22	6.44E-04	2.41E-03	hypothetical protein X797_007007
19259063	MAA_04777	1.5	1.9	3.5	4.1	5.9	5.9	-1.21	1.10E-02	2.87E-02	glucans biosynthesis glucosyltransferase H
19261992	MAA_07706	4.4	3.8	3.5	8.0	9.9	9.1	-1.20	3.73E-05	1.79E-04	major facilitator superfamily transporter
19259083	MAA_04797	34.9	43.7	36.8	68.5	97.2	100.0	-1.20	1.69E-08	1.36E-07	Epoxide hydrolase-like protein
19256065	MAA_01779	171.9	212.7	186.0	408.0	465.8	439.0	-1.20	6.81E-20	1.47E-18	EF-Hand 1, calcium-binding site
19262001	MAA_07715	97.4	105.6	91.6	196.0	255.6	225.2	-1.20	3.74E-14	5.09E-13	dienelactone hydrolase
19255988	MAA_01702	145.2	102.2	183.0	353.9	317.2	312.7	-1.19	6.55E-10	6.42E-09	Chloramphenicol acetyltransferase-like domain protein
19264981	MAA_10697	30.2	21.8	23.7	53.0	62.7	56.7	-1.19	1.52E-07	1.07E-06	HhH-GPD domain-containing protein
19256252	MAA_01966	12.5	17.8	12.4	31.5	32.5	32.7	-1.18	1.84E-08	1.48E-07	Amino acid/polyamine transporter I
19256264	MAA_01978	15.5	24.7	16.1	42.8	44.2	40.3	-1.18	5.52E-07	3.65E-06	hypothetical protein MAA_01978
19262019	MAA_07733	44.4	39.7	30.8	91.9	85.0	82.0	-1.17	3.13E-10	3.16E-09	lipase/thioesterase
19263121	MAA_08835	34.9	31.0	25.5	58.1	72.3	74.9	-1.17	1.86E-08	1.49E-07	von Willebrand factor, type A
19263663	MAA_09377	20.5	14.1	16.6	32.4	40.1	42.0	-1.16	1.57E-07	1.10E-06	SPFH domain/Band 7 family protein
19255901	MAA_01615	26.9	16.6	19.3	32.6	47.0	58.6	-1.14	4.22E-05	2.00E-04	glucose oxidase
19256650	MAA_02364	22.0	33.2	20.7	54.4	46.1	65.4	-1.13	1.40E-07	9.84E-07	ABC-2 type transporter
19264930	MAA_10645	38.1	38.5	44.8	58.2	100.0	106.6	-1.12	1.18E-05	6.06E-05	hypothetical protein MAA_10645
19256108	MAA_01822	122.2	114.4	110.0	238.0	283.1	234.3	-1.12	4.46E-18	8.62E-17	ribitol kinase
19258849	MAA_04563	87.2	87.0	76.3	144.7	214.1	184.6	-1.12	7.14E-11	7.78E-10	phosphoketolase, Mpk1
19258944	MAA_04658	384.3	375.4	459.0	861.2	949.3	816.5	-1.11	1.03E-20	2.35E-19	Cytochrome P450 CYP548A10
19256173	MAA_01887	117.9	132.6	126.3	262.4	279.7	269.6	-1.11	1.49E-29	6.06E-28	betaine aldehyde dehydrogenase
19256064	MAA_01778	20.6	19.5	25.0	46.5	44.2	49.4	-1.11	4.31E-10	4.31E-09	origin recognition complex subunit 6
19258798	MAA_04512	207.5	218.9	257.8	423.9	544.8	502.4	-1.10	9.81E-16	1.51E-14	PH domain containing protein
19258891	MAA_04605	35.0	45.1	33.5	75.5	88.6	79.1	-1.10	6.19E-11	6.86E-10	fungal specific transcription factor
19255914	MAA_01628	2.7	2.0	2.8	4.9	5.2	5.7	-1.09	7.14E-04	2.65E-03	Zn(2)-C6 fungal-type DNA-binding domain protein
19259249	MAA_04963	20.9	17.2	17.6	31.3	55.7	31.1	-1.09	4.33E-04	1.71E-03	SMP-30/Gluconolaconase/LRE-like region
19259014	MAA_04728	35.1	25.5	44.9	73.7	65.7	84.4	-1.08	4.04E-07	2.71E-06	hypothetical protein X797_001207
23632201	MAA_10752	23.9	16.9	14.0	43.8	36.0	36.1	-1.08	4.82E-05	2.24E-04	triacylglycerol lipase-like protein
19259016	MAA_04730	33.3	28.6	37.2	58.9	70.1	78.6	-1.07	1.70E-10	1.79E-09	glycoside hydrolase family 55
19264916	MAA_10630	28.2	29.3	31.7	53.5	78.5	54.6	-1.07	2.94E-06	1.69E-05	Zn(2)-Cys(6) zinc finger domain protein
19258971	MAA_04685	390.8	372.5	254.6	569.6	838.7	720.0	-1.06	1.48E-06	9.07E-06	hypothetical protein MAA_04685

19256021	MAA_01735	130.3	138.0	112.6	232.1	296.9	257.5	-1.05	2.20E-12	2.71E-11	histidine acid phosphatase
19259177	MAA_04891	17.0	15.8	11.6	28.6	32.8	29.9	-1.04	2.33E-06	1.38E-05	CoA-transferase family III
19259099	MAA_04813	419.6	410.0	437.7	786.1	929.3	887.6	-1.04	2.07E-20	4.61E-19	stress response RCI peptide
19256069	MAA_01783	128.0	106.9	97.3	238.4	226.0	215.1	-1.03	3.73E-13	4.76E-12	hypothetical protein MAA_01783
19262863	MAA_08577	10.5	6.6	8.7	16.8	19.6	16.2	-1.03	1.94E-04	8.17E-04	arginine metabolism regulation protein II
19259142	MAA_04856	403.1	356.9	392.2	684.8	910.3	748.3	-1.02	1.76E-13	2.32E-12	DUF323 domain protein
19259031	MAA_04745	296.7	361.1	234.8	549.6	609.6	628.8	-1.00	3.07E-09	2.74E-08	glucose transporter
19256171	MAA_01885	23.8	15.7	15.8	35.3	35.4	40.0	-1.00	9.10E-06	4.76E-05	amidase
19258997	MAA_04711	27.4	11.7	23.8	33.0	46.1	47.1	-1.00	3.04E-03	9.44E-03	PAP2 superfamily protein
19256626	MAA_02340	1369.7	1359.2	1397.3	803.9	583.0	639.8	1.03	3.15E-21	7.35E-20	Cytochrome P450 CYP541A4
19256543	MAA_02257	1218.8	1221.8	1457.2	637.0	644.7	602.5	1.05	4.56E-31	2.10E-29	DUF323 domain-containing protein
19256518	MAA_02232	122.3	106.4	129.1	61.5	50.4	61.0	1.05	1.01E-21	2.47E-20	hypothetical protein MAA_02232
19262598	MAA_08312	7353.4	7072.9	5661.1	3075.4	3391.0	3157.1	1.06	1.02E-19	2.15E-18	cell wall protein
19261981	MAA_07695	125.7	124.1	92.7	46.5	60.4	55.1	1.08	6.26E-10	6.17E-09	mitochondrial chaperone BCS1
19264983	MAA_10699	55.5	24.4	71.1	27.4	22.0	22.0	1.08	1.72E-03	5.72E-03	methyltransferase
19264970	MAA_10686	9853.3	7020.0	9760.5	4003.6	4485.7	4063.7	1.09	2.12E-16	3.41E-15	WD repeat containing protein 44
19258894	MAA_04608	226.6	210.9	215.0	101.2	102.8	100.9	1.10	1.58E-48	1.40E-46	proteinrelated to alpha-glucosidase b
19262600	MAA_08314	512.1	672.4	354.8	194.7	274.0	238.8	1.12	5.37E-08	3.97E-07	ATPase
19262592	MAA_08306	213.0	226.8	115.2	69.3	131.2	53.9	1.13	1.99E-02	4.83E-02	Ankyrin repeat protein
19259107	MAA_04821	8426.2	7870.8	8138.9	4071.9	3562.5	3530.3	1.13	1.31E-47	1.06E-45	hypothetical protein MAA_04821
19264969	MAA_10685	32.8	22.4	25.8	11.1	14.5	11.1	1.14	9.86E-08	7.07E-07	hypothetical protein MAA_10685
19258794	MAA_04508	162.7	153.7	168.7	75.1	65.3	77.6	1.15	3.93E-29	1.45E-27	Translation Initiation factor eIF- 4e-like domain protein
19256606	MAA_02320	359.8	294.6	278.0	121.7	141.5	150.6	1.17	7.10E-18	1.33E-16	hypothetical protein MAA_02320
19259211	MAA_04925	362.2	234.6	375.2	150.5	136.1	136.0	1.20	3.27E-13	4.20E-12	mitochondrial chaperone BCS1
19262030	MAA_07744	5.5	2.4	5.1	1.7	1.0	2.8	1.23	1.82E-02	4.49E-02	glucose-methanol-choline oxidoreductase
19259067	MAA_04781	34.6	25.2	32.4	9.4	16.0	13.3	1.25	5.95E-07	3.90E-06	hypothetical protein X797_001262
19256624	MAA_02338	1031.7	1043.2	999.0	502.9	400.0	374.0	1.27	9.22E-30	3.82E-28	General substrate transporter
19256423	MAA_02137	287.6	285.7	283.2	111.5	108.8	130.5	1.29	1.70E-37	1.12E-35	hypothetical protein MAA_02137
19259108	MAA_04822	4779.2	4026.0	5271.3	2129.3	1733.4	1866.8	1.30	6.68E-32	3.23E-30	Regulator of G protein signaling superfamily
19259240	MAA_04954	901.5	955.9	951.5	455.0	292.7	366.8	1.33	3.21E-24	9.17E-23	Cytochrome P450 CYP6001C17
19256584	MAA_02298	126.3	113.6	127.3	47.8	50.3	47.3	1.34	9.36E-53	9.04E-51	Signal transduction response regulator, receiver domain protein
19256537	MAA_02251	1105.7	960.5	926.8	482.4	364.5	334.9	1.34	4.15E-22	1.03E-20	chromosomal organization and DNA repair protein Mms21
19258920	MAA_04634	129.5	104.0	123.4	43.3	59.3	36.5	1.36	2.29E-10	2.35E-09	p-hydroxylaminobenzoate lyase

19263120	MAA_08834	86.9	76.5	92.4	33.9	33.1	32.4	1.36	7.60E-46	5.71E-44	multidrug resistance-associated protein 1
19256202	MAA_01916	678.7	584.5	484.5	214.9	228.3	222.3	1.39	3.20E-22	8.01E-21	hypothetical protein X797_000234
19262006	MAA_07720	10.6	26.4	12.2	6.2	6.7	5.2	1.44	3.19E-04	1.29E-03	hypothetical protein MAA_07720
19256694	MAA_02408	1321.1	1488.1	1540.1	615.0	519.8	462.2	1.45	1.51E-36	8.99E-35	Peptidase S8/S53, subtilisin/kexin/sedolisin
19255933	MAA_01647	40.7	46.5	51.1	17.0	15.3	18.3	1.45	3.13E-27	1.08E-25	protein kinase
19263164	MAA_08878	174.4	100.2	177.6	61.9	52.8	50.3	1.45	1.86E-13	2.44E-12	hypothetical protein MAA_08878
19259139	MAA_04853	457.5	529.5	327.0	164.0	158.1	146.5	1.49	1.27E-19	2.62E-18	hypothetical protein MAA_04853
19256744	MAA_02458	106.3	106.6	109.0	39.4	38.5	32.2	1.55	4.65E-25	1.39E-23	hypothetical protein MAA_02458
19256001	MAA_01715	448.4	200.1	398.7	131.7	109.7	102.1	1.61	1.75E-11	1.98E-10	DOMON domain protein
19262895	MAA_08609	2878.5	1498.2	2393.8	882.3	685.4	639.3	1.62	1.39E-15	2.10E-14	Alpha/beta hydrolase fold-3
19263648	MAA_09362	2771.5	2182.3	3248.5	901.3	785.3	721.4	1.77	1.46E-35	7.79E-34	SAM-dependent methyltransferase
19255990	MAA_01704	12.4	7.7	13.0	2.4	4.3	3.0	1.78	5.32E-06	2.92E-05	hypothetical protein MAA_01704
19258824	MAA_04538	155.9	175.0	186.9	53.3	43.0	50.5	1.82	6.99E-67	9.45E-65	CP2 transcription factor
19255996	MAA_01710	558.4	362.8	879.6	179.8	144.8	184.0	1.82	1.85E-07	1.28E-06	hypothetical protein MAA_01710
19262634	MAA_08348	2.3	3.8	2.7	0.5	1.1	0.7	1.96	4.28E-03	1.29E-02	phytanoyl-CoA dioxygenase
19262891	MAA_08605	5.9	6.8	7.3	1.0	3.5	0.2	2.08	5.42E-03	1.57E-02	hypothetical protein MAA_08605
19262913	MAA_08627	7.1	7.4	8.0	1.5	1.0	2.4	2.18	1.24E-05	6.35E-05	hypothetical protein MAA_08627
19256536	MAA_02250	14168.7	12149.4	16851.2	3462.8	2773.7	2657.6	2.28	5.02E-64	6.37E-62	PAS domain containing protein
19256328	MAA_02042	1989.2	1311.1	1488.6	397.5	306.7	277.3	2.29	6.26E-40	4.38E-38	3-oxo-5-alpha-steroid 4-dehydrogenase
19256607	MAA_02321	250.6	141.7	384.9	49.5	56.3	47.5	2.34	6.05E-18	1.15E-16	Major facilitator superfamily domain, general substrate transporter
19263645	MAA_09359	14.6	17.3	17.7	3.3	1.8	3.4	2.54	6.72E-30	2.84E-28	Pfs, NACHT and WD domain protein
19259066	MAA_04780	21.8	17.2	19.4	2.2	5.5	1.8	2.60	9.72E-10	9.35E-09	hypothetical protein MAA_04780
19259065	MAA_04779	7.2	8.1	11.6	0.8	1.0	0.8	3.37	9.28E-23	2.45E-21	hypothetical protein MAA_04779
19263644	MAA_09358	2554.8	3029.2	3175.7	302.8	245.0	252.1	3.45	3.70E-209	2.50E-206	hypothetical protein MAA_09358
19256297	MAA_02011	31.1	20.1	26.5	2.1	2.2	0.9	3.91	2.90E-17	5.11E-16	hypothetical protein MAA_02011
19263643	MAA_09357	931.7	966.2	1214.5	73.1	47.3	48.8	4.20	5.62E-142	2.28E-139	hypothetical protein X797_010135
19263642	MAA_09356	6633.0	7477.7	8392.6	549.2	325.8	338.3	4.21	3.54E-123	7.98E-121	hemeolysin E
19264933	MAA_10648	11.1	0.0	8.5	0.0	0.0	0.0	16.00	8.34E-03	2.30E-02	ATPase domain, prokaryote

Table S3. A list of differentially expressed genes identified from the Δ*abaA*-1 mutant versus the WT strain of *M. roberstii*.

Gene_id	Gene_name	FPKM			FPKM			Δ*abaA*/WT			Description
		Δ*abaA*_1	Δ*abaA*_2	Δ*abaA*_3	WT_1	WT_2	WT_3	log2 *R*	*p*-value	*q*-value	
19256296	MAA_02010	0.0	0.0	0.0	4.4	6.8	4.7	-15.69	3.52E-12	3.94E-11	Amino acid transporter, transmembrane
19262897	MAA_08611	0.0	0.0	0.0	4.6	5.9	3.7	-15.53	6.75E-09	5.04E-08	hypothetical protein X797_008967
19255911	MAA_01625	0.0	0.0	0.0	1.1	1.3	0.7	-13.34	1.85E-03	5.67E-03	Thioesterase
19262625	MAA_08339	1.1	0.0	10.9	7189.7	11527.9	8575.0	-11.15	5.39E-12	5.74E-11	hypothetical protein MAA_08339
19263637	MAA_09351	15.6	31.8	65.1	48866.7	83933.1	58904.2	-10.73	7.36E-70	8.27E-68	hypothetical protein MAA_09351
19259048	MAA_04762	0.7	1.3	2.1	622.7	1036.0	809.9	-9.19	4.38E-90	7.38E-88	Multicopper oxidase, copper-binding site
19262594	MAA_08308	0.1	0.0	0.0	17.9	28.0	19.4	-9.18	8.32E-17	1.37E-15	Peptidase aspartic, active site protein
19259151	MAA_04865	0.4	0.0	0.6	127.5	195.1	109.4	-8.85	5.11E-42	2.35E-40	Peptidoglycan-binding lysin domain protein
19264886	MAA_10600	0.2	0.0	0.0	5.1	5.4	1.5	-6.30	4.26E-07	2.48E-06	proteinrelated to zinc finger protein odd-paired-like (opl)
19264873	MAA_10587	0.1	0.0	0.0	0.0	1.7	1.1	-5.55	8.50E-03	2.15E-02	O-methyltransferase, family 2
19264888	MAA_10602	0.6	0.0	0.0	7.1	9.7	11.9	-5.55	4.28E-07	2.48E-06	subtilisin-like protease
19258736	MAA_04450	14.2	13.6	9.2	508.7	612.8	408.8	-5.37	2.61E-185	1.32E-182	Glycoside hydrolase, superfamily
19262028	MAA_07742	0.2	0.0	0.0	2.3	5.5	0.4	-5.36	2.24E-04	8.10E-04	subtilisin-like protease
19264949	MAA_10665	44.5	43.7	59.0	1578.2	1999.0	1677.9	-5.16	2.00E-303	5.00E-300	hypothetical protein X797_001079
19258931	MAA_04645	7.1	11.1	17.1	238.8	548.6	458.8	-5.14	1.53E-27	4.26E-26	GPR1/FUN34/yaaH family protein
19263638	MAA_09352	0.4	0.7	1.0	14.0	30.8	28.1	-5.08	1.35E-28	4.09E-27	hypothetical protein MAA_09352
19255910	MAA_01624	0.0	0.0	0.2	1.0	2.2	1.8	-4.96	8.74E-05	3.45E-04	LysR family regulatory protein
19255960	MAA_01674	0.9	1.6	1.3	18.6	42.7	25.5	-4.53	2.18E-16	3.48E-15	Endoribonuclease L-PSP/chorismate mutase-like protein
19261999	MAA_07713	0.7	3.9	1.0	20.9	51.3	27.5	-4.16	2.59E-12	2.98E-11	hypothetical protein MAA_07713
19262633	MAA_08347	0.0	0.4	0.0	1.9	1.7	2.7	-4.10	1.20E-03	3.79E-03	phytanoyl-CoA dioxygenase
19262627	MAA_08341	1.1	0.0	0.3	4.2	11.6	4.6	-3.84	1.15E-06	6.34E-06	hypothetical protein MAA_08341
19262027	MAA_07741	129.6	134.9	97.3	1583.4	1903.4	1664.4	-3.83	2.24E-187	1.51E-184	Formate/nitrite transporter
19262899	MAA_08613	166.2	195.5	197.7	1995.8	2517.0	1934.8	-3.53	3.29E-162	1.33E-159	ntf2-like domain protein
19263092	MAA_08806	2.1	1.8	4.7	34.4	39.2	21.4	-3.44	1.67E-21	3.59E-20	Aminoglycoside phosphotransferase
19263635	MAA_09349	1.2	1.0	0.6	11.7	8.5	8.3	-3.37	2.37E-19	4.44E-18	hypothetical protein X797_010127
19256738	MAA_02452	1.7	0.5	0.0	6.2	7.7	8.7	-3.33	6.87E-05	2.78E-04	alcohol dehydrogenase
19256645	MAA_02359	440.9	456.6	437.2	4512.0	4602.1	3591.3	-3.25	2.07E-198	2.10E-195	Carbohydrate-binding WSC
19255923	MAA_01637	1.8	3.1	0.8	5.8	18.9	28.4	-3.20	1.37E-07	8.62E-07	hypothetical protein MAA_01637
19259312	MAA_05026	36.5	40.3	45.6	331.4	404.1	332.5	-3.13	3.37E-118	8.53E-116	galactosyltransferase superfamily protein

19255920	MAA_01634	0.8	0.0	0.0	0.5	3.7	2.4	-3.09	1.64E-02	3.81E-02	aminoglycoside 3'-phosphotransferase/choline kinase domain protein
19256662	MAA_02376	14.8	19.3	23.5	136.5	202.9	149.7	-3.09	2.22E-56	1.73E-54	Aromatic-ring hydroxylase-like protein
19258964	MAA_04678	446.4	338.2	210.8	2956.7	2508.1	2711.8	-3.04	1.40E-24	3.35E-23	ATPase, F0 complex, subunit C, DCCD-binding site
19262004	MAA_07718	1.8	2.6	0.0	5.5	18.1	12.6	-3.04	1.00E-03	3.19E-03	hypothetical protein MAA_07718
19255951	MAA_01665	0.0	0.2	0.2	1.9	0.8	0.7	-3.02	4.72E-03	1.29E-02	collagen-like protein Mcl1
19263091	MAA_08805	4.6	2.2	3.6	22.9	33.2	22.1	-2.90	9.73E-11	8.95E-10	IDI-2 precursor
19256187	MAA_01901	6.6	3.8	7.6	40.3	50.7	37.7	-2.83	2.65E-20	5.21E-19	DUF967 domain protein
19263636	MAA_09350	0.7	1.5	0.4	6.7	6.7	5.2	-2.83	5.67E-12	6.01E-11	hypothetical protein MAA_09350
19262656	MAA_08370	5.2	8.2	1.0	23.6	41.8	35.7	-2.82	2.24E-04	8.10E-04	polyketide synthase
19259129	MAA_04843	12.1	11.2	13.1	74.9	94.0	71.8	-2.73	1.87E-66	2.00E-64	Basic-leucine zipper (bZIP) transcription factor
19264993	MAA_10709	6.7	7.4	5.6	43.2	45.7	38.1	-2.68	4.63E-56	3.35E-54	nicotinamide N-methyltransferase
19255928	MAA_01642	0.8	2.5	0.4	2.5	7.9	12.7	-2.68	4.18E-03	1.16E-02	fatty acid synthase subunit alpha reductase
19264986	MAA_10702	1.9	2.9	0.7	12.2	11.5	11.2	-2.66	2.79E-11	2.81E-10	D-xylulose reductase A
19256325	MAA_02039	44.7	51.2	31.9	239.7	298.2	228.3	-2.58	8.67E-47	5.02E-45	Glutathione S-transferase/chloride channel
19258976	MAA_04690	2.5	5.7	3.5	15.6	24.0	29.7	-2.57	1.24E-14	1.73E-13	DUF1479 domain protein
19258823	MAA_04537	12.7	9.9	11.6	65.8	74.1	61.5	-2.56	2.22E-63	2.14E-61	Major facilitator superfamily domain protein
19256699	MAA_02413	117.3	91.6	46.9	551.1	424.9	499.3	-2.53	1.02E-12	1.23E-11	C4-dicarboxylate transporter/malic acid transporter
19256169	MAA_01883	27.0	23.8	20.9	129.0	139.0	128.3	-2.47	1.66E-109	3.73E-107	ABC-2 type transporter
19256403	MAA_02117	84.3	92.5	84.4	531.4	454.0	459.4	-2.47	1.90E-134	6.41E-132	Short-chain dehydrogenase/reductase SDR
19263676	MAA_09390	132.2	116.1	135.6	709.1	784.2	611.8	-2.46	1.55E-78	2.24E-76	putative protein family Cys-rich
23632178	MAA_10729	4.6	8.7	1.8	25.5	36.7	20.1	-2.45	1.54E-04	5.76E-04	hypothetical protein MAA_10729
19255925	MAA_01639	1.3	3.6	1.2	3.5	8.0	18.4	-2.31	3.65E-03	1.04E-02	peptide synthetase
19256017	MAA_01731	246.2	228.8	282.4	1123.5	1211.9	964.7	-2.12	4.08E-77	5.51E-75	multicopper oxidase
19256766	MAA_02480	21.8	25.6	26.6	104.8	120.2	96.9	-2.12	6.18E-63	5.21E-61	NAD-specific glutamate dehydrogenase
19262572	MAA_08286	3.1	2.3	3.6	8.6	19.1	11.3	-2.11	1.55E-07	9.70E-07	dehydrogenase/reductase SDR family
19256307	MAA_02021	12.0	3.2	6.5	25.2	39.8	25.1	-2.05	9.72E-06	4.57E-05	hypothetical protein MAA_02021
19262592	MAA_08306	36.6	20.5	5.2	69.3	131.2	53.9	-2.03	1.06E-02	2.63E-02	Ankyrin repeat protein
19259268	MAA_04982	7.9	10.8	7.3	32.8	35.1	36.9	-2.01	3.35E-27	8.92E-26	protein-arginine deiminase type-4
19255921	MAA_01635	3.1	6.3	1.0	10.6	14.2	14.5	-1.92	1.22E-04	4.66E-04	integral membrane protein pth11
19256739	MAA_02453	26.2	23.8	24.7	76.6	114.5	83.3	-1.88	1.67E-33	6.05E-32	arrestin domain-containing protein
19256386	MAA_02100	132.6	153.9	98.0	451.1	450.7	486.8	-1.85	9.93E-39	4.37E-37	branched chain aminotransferase
19258999	MAA_04713	28.7	26.3	20.3	60.1	116.4	95.0	-1.85	3.30E-12	3.73E-11	hypothetical protein MAA_04713
19259036	MAA_04750	18.7	12.3	9.5	36.6	55.4	52.9	-1.83	2.42E-10	2.10E-09	

19264926	MAA_10641	156.3	203.4	177.2	630.6	652.9	577.2	-1.79	3.82E-48	2.35E-46	carbohydrate esterase family 1 protein
19255930	MAA_01644	90.4	147.9	50.9	126.2	301.6	543.2	-1.75	8.59E-03	2.16E-02	RTA-like protein
19255939	MAA_01653	1.9	7.6	3.5	6.4	13.4	23.7	-1.74	4.93E-03	1.33E-02	hypothetical protein MAA_01653
19262912	MAA_08626	2.1	1.5	1.0	6.5	5.0	3.8	-1.74	5.00E-04	1.70E-03	Protein kinase-like protein
19256313	MAA_02027	5.8	8.0	5.9	15.0	26.4	22.2	-1.69	4.45E-08	3.02E-07	hypothetical protein MAA_02027
23632179	MAA_10730	27.6	37.9	29.9	82.4	129.8	93.5	-1.68	3.46E-15	5.12E-14	Bromodomain protein
19262613	MAA_08327	140.1	114.3	108.8	329.4	464.6	352.9	-1.66	6.23E-29	1.91E-27	hypothetical protein MAA_08327
19263129	MAA_08843	7.5	5.2	3.3	8.9	16.7	24.5	-1.64	1.38E-02	3.27E-02	hypothetical protein MAA_08843
19256663	MAA_02377	20.2	20.6	24.6	61.8	53.3	85.8	-1.62	1.35E-24	3.30E-23	ATP-binding cassette transporter ifT1
19262014	MAA_07728	75.4	83.6	80.2	227.0	266.7	236.2	-1.61	2.71E-52	1.83E-50	transmembrane amino acid transporter
19255924	MAA_01638	3.4	6.8	5.3	6.1	12.5	27.5	-1.57	1.28E-02	3.09E-02	canalicular multispecific organic anion transporter 1
19256685	MAA_02399	104.8	152.2	129.3	337.9	313.3	478.8	-1.55	1.19E-22	2.70E-21	ATPase, P-type phosphorylation site
19256213	MAA_01927	3.1	1.6	2.2	6.0	7.4	7.0	-1.55	2.65E-04	9.42E-04	hypothetical protein MAA_01927
19263667	MAA_09381	25.3	28.8	10.5	43.7	76.0	69.0	-1.55	3.31E-06	1.69E-05	protoporphyrinogen oxidase
19258970	MAA_04684	266.1	175.1	158.5	570.9	584.9	570.6	-1.53	1.49E-20	2.96E-19	arginase-like protein
19256376	MAA_02090	98.7	89.0	107.1	236.9	338.9	261.1	-1.51	1.63E-22	3.66E-21	GCN5-related N-acetyltransferase (GNAT) domain protein
19256065	MAA_01779	141.8	141.0	182.4	408.0	465.8	439.0	-1.50	1.34E-32	4.78E-31	EF-Hand 1, calcium-binding site
19258785	MAA_04499	134.5	134.0	127.6	299.6	441.2	362.5	-1.48	2.25E-28	6.61E-27	pyruvate decarboxylase
19259303	MAA_05017	2460.7	2701.3	2098.2	5408.9	7974.6	6431.8	-1.45	7.17E-25	1.77E-23	Extracellular membrane protein, CFEM domain protein
19256318	MAA_02032	19.5	19.6	18.7	47.6	63.1	43.9	-1.42	4.70E-16	7.22E-15	3-phytase
19256335	MAA_02049	112.1	125.7	101.4	295.4	277.6	325.4	-1.41	2.64E-32	9.20E-31	hypothetical protein MAA_02049
23632205	MAA_10756	38.6	38.0	46.8	138.2	89.8	97.5	-1.40	9.49E-15	1.35E-13	hypothetical protein MAA_10756
19259267	MAA_04981	10.8	10.3	11.5	29.1	31.4	25.2	-1.40	4.18E-13	5.16E-12	Extracellular membrane protein, 8-cysteine region, CFEM
19262896	MAA_08610	1.5	1.1	1.6	3.0	4.6	3.2	-1.34	3.91E-03	1.10E-02	Protein kinase domain containing protein
19255927	MAA_01641	17.9	21.4	18.4	30.6	41.4	71.8	-1.32	9.44E-07	5.28E-06	ankyrin repeat protein
19259074	MAA_04788	57.4	76.1	47.0	113.4	178.6	154.2	-1.31	5.00E-10	4.14E-09	dopa 4,5-dioxygenase
19256283	MAA_01997	26.2	30.8	24.8	64.9	74.5	62.6	-1.30	3.59E-18	6.25E-17	Carboxylesterase type B, active site protein
19255915	MAA_01629	8.1	5.8	6.1	12.8	15.1	21.3	-1.30	2.30E-04	8.28E-04	GCN5-related N-acetyltransferase (GNAT) domain protein
19263640	MAA_09354	2.2	2.6	2.4	4.0	8.1	5.5	-1.29	1.82E-02	4.13E-02	C6 transcription factor (Acr-2)
19262898	MAA_08612	130.3	131.7	113.4	294.3	352.2	269.7	-1.29	1.45E-20	2.90E-19	flavodoxin-like protein
19263633	MAA_09347	5.5	10.1	6.6	15.8	22.7	15.2	-1.28	1.24E-04	4.72E-04	transmembrane amino acid transporter
19258968	MAA_04682	152.2	108.5	84.2	273.7	265.5	295.6	-1.28	5.81E-14	7.65E-13	hypothetical protein MAA_04682
19264966	MAA_10682	21.0	18.7	14.4	35.5	42.6	52.7	-1.27	2.63E-12	3.01E-11	WD40 domain protein

19262017	MAA_07731	56.1	56.6	56.1	135.8	144.9	123.5	-1.26	1.97E-27	5.31E-26	integral membrane protein
19264975	MAA_10691	5.9	5.6	10.0	15.0	18.6	16.8	-1.23	1.72E-05	7.83E-05	S-adenosyl-L-methionine-dependent methyltransferase
19262582	MAA_08296	30.4	27.0	44.6	92.3	64.3	80.7	-1.22	3.46E-06	1.75E-05	phosphotransferase
19256511	MAA_02225	110.2	126.4	114.9	278.7	273.4	263.8	-1.22	5.99E-43	2.82E-41	electron transfer flavoprotein
19256693	MAA_02407	17.8	22.1	17.8	49.1	44.1	40.2	-1.21	1.49E-12	1.75E-11	Bicupin, oxalate decarboxylase/oxidase
19259153	MAA_04867	6.6	7.4	4.5	15.2	13.5	14.0	-1.21	2.92E-06	1.50E-05	Pyridine nucleotide-disulfide oxidoreductase
19256036	MAA_01750	425.7	516.4	421.9	1068.2	1064.4	1004.6	-1.20	6.60E-32	2.23E-30	bacterial-type extracellular deoxyribonuclease
19262856	MAA_08570	120.3	111.6	146.7	225.4	374.2	270.5	-1.20	1.07E-10	9.74E-10	glyoxalase family protein
19256221	MAA_01935	25.0	32.7	18.2	44.4	58.0	70.6	-1.19	2.57E-08	1.80E-07	integral membrane protein
19259195	MAA_04909	68.5	74.2	73.4	133.5	185.0	167.0	-1.17	3.06E-18	5.38E-17	major facilitator superfamily transporter
19259203	MAA_04917	38.1	45.9	31.9	85.2	101.1	71.2	-1.15	1.21E-06	6.64E-06	Stress responsive alpha-beta barrel
19258996	MAA_04710	155.7	146.3	62.9	171.3	319.4	312.5	-1.14	1.41E-02	3.35E-02	Glutathione S-transferase/chloride channel
19265003	MAA_10719	57.9	57.3	82.5	158.9	111.8	163.5	-1.14	4.58E-11	4.50E-10	YTH domain protein
19258971	MAA_04685	347.5	358.1	268.5	569.6	838.7	720.0	-1.13	1.74E-12	2.03E-11	hypothetical protein MAA_04685
19259152	MAA_04866	48.9	70.3	64.5	120.5	148.7	129.0	-1.12	4.95E-12	5.33E-11	SAM-dependent methyltransferase
19256469	MAA_02183	2495.5	2680.1	4165.8	4145.2	9244.2	6771.9	-1.11	4.69E-03	1.28E-02	Glucose-repressible protein Grg1
19261930	MAA_07644	226.4	213.4	248.2	423.1	540.6	513.6	-1.10	2.89E-21	6.10E-20	hypothetical protein MAA_07644
19262026	MAA_07740	11.1	18.5	10.2	23.9	36.5	24.8	-1.10	6.41E-05	2.62E-04	muconate cycloisomerase I, MLE
19263682	MAA_09396	381.7	416.6	416.2	763.4	971.8	804.9	-1.06	1.89E-22	4.22E-21	Alcohol dehydrogenase superfamily, zinc-type
19263639	MAA_09353	34.8	27.8	21.1	49.9	62.5	62.1	-1.06	2.62E-07	1.58E-06	hypothetical protein MAA_09353
19256239	MAA_01953	4.8	5.4	4.8	9.7	10.0	11.2	-1.05	1.28E-04	4.86E-04	dipeptidyl aminopeptidase/acylaminoacyl peptidase
19259294	MAA_05008	10.2	8.4	6.4	16.3	18.1	16.7	-1.04	2.93E-05	1.27E-04	taurine catabolism dioxygenase TauD
19258994	MAA_04708	46.3	59.1	34.0	94.0	87.6	104.0	-1.03	4.32E-09	3.29E-08	Major facilitator superfamily domain, general substrate transporter
19256130	MAA_01844	118.0	85.3	115.8	233.0	201.4	215.5	-1.03	2.16E-13	2.70E-12	cytochrome oxidase assembly
19259233	MAA_04947	7.6	7.6	5.1	14.1	12.9	14.3	-1.02	5.53E-03	1.47E-02	DSBA-like thioredoxin domain-containing protein
19256013	MAA_01727	17.2	12.3	13.2	23.6	26.0	36.4	-1.01	1.78E-04	6.56E-04	meiosis protein SPO22/ZIP4 like protein
19256285	MAA_01999	152.1	135.7	152.4	289.8	288.0	306.5	-1.01	9.57E-28	2.69E-26	AoPex11B-like protein
19264954	MAA_10670	183.9	208.1	168.1	53.2	96.5	130.2	1.00	1.62E-04	6.05E-04	hypothetical protein MAA_10670
19262604	MAA_08318	227.4	233.1	274.9	102.9	135.2	127.9	1.01	1.53E-14	2.09E-13	pH-response regulator protein palA/RIM20
19261966	MAA_07680	536.1	619.2	635.3	308.9	276.3	304.0	1.01	3.00E-22	6.53E-21	hypothetical protein MAA_07680
19263130	MAA_08844	8.7	10.5	6.1	3.7	2.8	6.1	1.01	1.44E-02	3.39E-02	ATP-grasp fold protein
19263683	MAA_09397	6178.5	6560.2	6182.2	3023.7	2969.9	3291.1	1.03	4.99E-44	2.41E-42	hypothetical protein MAA_09397
19258791	MAA_04505	383.4	409.8	362.2	215.1	162.1	183.3	1.04	6.66E-19	1.23E-17	negative acting factor-like protein

19256660	MAA_02374	140.5	162.2	210.4	78.8	79.6	89.8	1.05	1.88E-12	2.18E-11	U-box domain containing protein
19256608	MAA_02322	1553.0	979.2	1147.9	475.0	792.1	508.1	1.05	2.18E-03	6.54E-03	copper resistance protein Crd2, partial
19256472	MAA_02186	86.3	94.4	107.0	42.7	46.3	49.4	1.06	9.56E-18	1.63E-16	acetyl-hydrolase
19256621	MAA_02335	154.1	121.1	132.8	61.7	63.6	70.8	1.06	6.27E-17	1.04E-15	glycoside hydrolase family 1 protein
19264907	MAA_10621	111.9	108.1	110.8	51.1	53.0	52.0	1.08	4.67E-26	1.20E-24	MYB DNA-binding domain-containing protein
19259257	MAA_04971	61.1	64.3	61.0	33.7	27.4	26.8	1.08	4.59E-05	1.92E-04	hypothetical protein MAA_04971
19258828	MAA_04542	2395.3	2199.8	2305.5	1151.5	1036.6	1065.5	1.08	1.62E-45	8.63E-44	Peptidase S10, serine carboxypeptidase
19259248	MAA_04962	141.1	137.5	170.2	68.2	70.3	72.2	1.09	4.24E-20	8.26E-19	aldehyde dehydrogenase
19256328	MAA_02042	511.6	558.8	1021.8	397.5	306.7	277.3	1.09	4.61E-03	1.27E-02	3-oxo-5-alpha-steroid 4-dehydrogenase
19256298	MAA_02012	88.8	94.8	95.8	45.5	36.2	49.2	1.09	1.37E-13	1.73E-12	Arv1-like family protein
19259155	MAA_04869	7.1	6.0	9.2	2.6	2.0	5.7	1.10	1.76E-02	4.02E-02	Glycoside hydrolase, clan GH-D
19263120	MAA_08834	81.6	62.4	70.4	33.9	33.1	32.4	1.11	1.27E-20	2.57E-19	multidrug resistance-associated protein 1
19262580	MAA_08294	71.2	60.3	68.3	26.6	35.0	31.0	1.11	2.12E-11	2.18E-10	Aminoglycoside phosphotransferase
19264965	MAA_10681	30.0	31.5	26.2	8.7	17.8	14.1	1.11	7.30E-05	2.93E-04	hypothetical protein MAA_10681
19255988	MAA_01702	725.6	739.0	673.9	353.9	317.2	312.7	1.12	6.37E-36	2.53E-34	Chloramphenicol acetyltransferase-like domain protein
19259172	MAA_04886	265.2	247.2	290.8	129.2	113.5	124.7	1.13	1.03E-25	2.60E-24	hypothetical protein MAA_04886
19261979	MAA_07693	17.8	14.5	14.1	8.1	7.6	5.4	1.13	3.57E-05	1.52E-04	beta-xylosidase
19261902	MAA_07616	204.9	221.9	188.1	88.3	93.9	97.4	1.14	3.75E-28	1.09E-26	Low temperature requirement A
19264908	MAA_10622	1660.4	1849.3	1733.7	825.3	778.5	780.8	1.14	4.00E-46	2.25E-44	hypothetical protein MAA_10622
19259304	MAA_05018	63.4	79.2	73.5	23.9	32.8	40.2	1.16	2.41E-07	1.47E-06	hypothetical protein MAA_05018
19263632	MAA_09346	135.5	117.8	183.0	60.3	66.4	66.6	1.17	1.14E-11	1.19E-10	polysaccharide deacetylase
19256174	MAA_01888	489.8	480.1	513.7	206.0	221.7	222.7	1.19	4.27E-48	2.55E-46	choline oxidase
19256043	MAA_01757	354.0	354.3	364.0	150.4	156.0	160.4	1.20	3.61E-44	1.78E-42	hypothetical protein MAA_01757
19256518	MAA_02232	127.2	124.4	148.1	61.5	50.4	61.0	1.21	3.48E-25	8.69E-24	hypothetical protein MAA_02232
19259107	MAA_04821	7472.8	8504.6	10125.9	4071.9	3562.5	3530.3	1.23	1.41E-24	3.35E-23	hypothetical protein MAA_04821
19264900	MAA_10614	16.3	18.7	16.2	8.4	6.8	6.4	1.25	1.69E-07	1.05E-06	impB/mucB/samB family protein
19261903	MAA_07617	1524.7	1672.0	1585.4	686.4	659.7	660.9	1.25	3.51E-56	2.63E-54	hypothetical protein MAA_07617
19255993	MAA_01707	12.6	14.8	15.6	5.5	6.4	6.1	1.25	7.57E-10	6.09E-09	hypothetical protein MAA_01707
19259247	MAA_04961	118.4	105.1	74.9	43.8	46.0	34.6	1.26	7.41E-11	6.98E-10	vacuolar triacylglycerol lipase
19255955	MAA_01669	21.8	17.1	18.2	5.9	7.0	10.8	1.27	4.29E-05	1.80E-04	Protein kinase-like protein
19259086	MAA_04800	911.8	958.0	1159.7	506.0	378.8	366.3	1.28	7.85E-20	1.49E-18	Glucose receptor Git3
19264961	MAA_10677	90.5	92.2	101.6	38.5	37.4	41.5	1.28	3.70E-32	1.27E-30	hypothetical protein MAA_10677
19256001	MAA_01715	239.6	262.4	335.4	131.7	109.7	102.1	1.29	2.63E-17	4.44E-16	DOMON domain protein

19264983	MAA_10699	55.6	59.9	61.7	27.4	22.0	22.0	1.31	4.56E-11	4.50E-10	methyltransferase
19264970	MAA_10686	10118.0	10153.0	11221.5	4003.6	4485.7	4063.7	1.33	3.60E-49	2.28E-47	WD repeat containing protein 44
19259066	MAA_04780	5.5	7.1	11.5	2.2	5.5	1.8	1.33	1.36E-02	3.24E-02	hypothetical protein MAA_04780
19258947	MAA_04661	291.4	274.0	304.6	117.7	114.6	112.2	1.34	1.98E-55	1.39E-53	Ubiquitin system component Cue
19263096	MAA_08810	33.2	37.9	42.7	14.0	15.0	15.6	1.35	1.08E-26	2.84E-25	ATPase-like, ATP-binding domain protein
19255953	MAA_01667	47.7	50.5	73.3	21.2	22.5	22.7	1.37	1.28E-14	1.78E-13	Transcription factor, fungi
19255986	MAA_01700	552.4	615.4	558.0	233.2	234.8	193.9	1.38	8.81E-37	3.57E-35	protein-arginine deiminase type-4
19258907	MAA_04621	3028.1	3071.0	3837.9	1289.7	1381.8	1133.6	1.38	6.50E-30	2.06E-28	hypothetical protein MAA_04621
19258894	MAA_04608	278.6	236.9	292.9	101.2	102.8	100.9	1.41	2.70E-44	1.40E-42	proteinrelated to alpha-glucosidase b
19255992	MAA_01706	3.3	2.6	1.9	0.7	1.0	1.2	1.41	1.78E-02	4.06E-02	hypothetical protein MAA_01706
19258898	MAA_04612	22.5	21.9	28.3	7.4	9.6	9.6	1.45	4.23E-12	4.64E-11	Mg^{2+} transporter protein, CorA-like/Zinc transport protein ZntB
19259256	MAA_04970	44.7	60.8	39.2	17.5	20.4	14.6	1.46	1.73E-06	9.25E-06	hypothetical protein MAA_04970
19261978	MAA_07692	17.6	18.0	33.7	10.9	4.0	10.2	1.46	2.56E-04	9.12E-04	carbohydrate-binding protein, partial
19264882	MAA_10596	349.6	360.4	320.5	129.7	119.0	123.4	1.47	3.12E-63	2.87E-61	hypothetical protein MAA_10596
19259266	MAA_04980	451.4	518.9	722.6	175.2	185.8	239.8	1.49	2.06E-18	3.65E-17	extracellular dioxygenase
19255913	MAA_01627	20.0	30.1	22.0	9.8	9.8	5.6	1.52	6.40E-06	3.10E-05	L-xylulose reductase
19263628	MAA_09342	19.0	16.5	26.5	1.5	5.5	14.7	1.52	1.46E-02	3.44E-02	carbohydrate-binding module family 13 protein
19264885	MAA_10599	3434.9	3322.6	3437.2	1348.0	1116.6	1019.5	1.55	1.15E-45	6.31E-44	leucine rich repeat domain containing protein
19261998	MAA_07712	406.0	380.6	491.7	141.9	145.6	145.9	1.56	2.37E-37	1.00E-35	hypothetical protein MAA_07712
19256314	MAA_02028	5.6	7.4	7.2	3.9	1.3	1.6	1.57	2.03E-03	6.13E-03	cyclopropane-fatty-acyl-phospholipid synthase
19258829	MAA_04543	820.3	904.0	736.5	253.6	296.6	273.5	1.58	4.74E-41	2.13E-39	Phospholipase A2
19255989	MAA_01703	261.3	247.0	257.5	99.1	73.3	79.5	1.60	6.87E-38	2.96E-36	zinc finger domain-containing protein
19262903	MAA_08617	63.5	51.0	70.2	27.8	16.7	14.9	1.64	4.81E-12	5.20E-11	aromatic amino acid aminotransferase
19256453	MAA_02167	123.2	142.4	186.7	46.9	48.3	49.2	1.65	5.03E-24	1.18E-22	Glucoamylase, putative
19255987	MAA_01701	385.0	346.6	354.7	133.5	120.9	90.7	1.65	5.43E-34	2.00E-32	polyketide synthase
19256223	MAA_01937	34.8	45.0	37.4	9.8	11.8	14.0	1.72	9.24E-17	1.50E-15	O-methyltransferase, family 2
19263645	MAA_09359	11.1	9.3	8.3	3.3	1.8	3.4	1.76	3.84E-12	4.25E-11	Pfs, NACHT and WD domain protein
19256461	MAA_02175	580.1	510.7	635.5	184.6	152.6	167.3	1.77	3.61E-63	3.17E-61	immunoglobulin I-set domain-containing protein
19256537	MAA_02251	1336.1	1347.9	1841.9	482.4	364.5	334.9	1.94	4.58E-35	1.75E-33	chromosomal organization and DNA repair protein Mms21
19261904	MAA_07618	1851.2	1923.4	2135.9	518.8	524.5	481.3	1.95	2.73E-104	5.52E-102	cytochrome P450
19256694	MAA_02408	2259.2	1895.8	2119.2	615.0	519.8	462.2	1.97	4.27E-62	3.46E-60	Peptidase S8/S53, subtilisin/kexin/sedolisin
19262576	MAA_08290	50.4	69.1	53.1	9.2	19.1	15.2	1.99	4.59E-12	5.00E-11	hypothetical protein MAA_08290
19255932	MAA_01646	24.4	18.3	21.7	4.8	4.0	7.4	1.99	1.13E-13	1.45E-12	Tyrosine-protein kinase, active site protein

19264977	MAA_10693	39.0	55.4	45.6	11.8	11.7	10.9	2.02	3.61E-18	6.25E-17	hypothetical protein MAA_10693
19263644	MAA_09358	1476.0	990.9	850.5	302.8	245.0	252.1	2.05	1.76E-31	5.84E-30	hypothetical protein MAA_09358
19262909	MAA_08623	524.2	474.5	668.6	165.8	106.4	127.7	2.06	3.93E-35	1.53E-33	phytanoyl-CoA dioxygenase
19264909	MAA_10623	1925.3	2056.7	2255.7	515.6	464.6	477.4	2.10	1.26E-124	3.65E-122	DNA-binding WRKY domain-containing protein
19263139	MAA_08853	4.1	3.3	7.7	0.9	1.3	1.0	2.25	7.69E-06	3.65E-05	Cytochrome P450 CYP540B20
19262907	MAA_08621	31.2	23.1	31.1	6.1	3.9	6.9	2.34	9.04E-16	1.36E-14	f420-dependent NADP reductase
19263162	MAA_08876	275.5	284.6	402.3	69.7	66.2	53.8	2.34	3.03E-44	1.53E-42	DNA double-strand break repair and VJ recombination XRCC4
19258948	MAA_04662	515.5	402.8	550.8	95.4	93.5	99.8	2.35	5.51E-90	8.59E-88	zinc finger domain-containing protein
19256607	MAA_02321	280.8	288.0	246.7	49.5	56.3	47.5	2.41	2.54E-91	4.68E-89	Major facilitator superfamily domain, general substrate transporter
19256536	MAA_02250	12775.9	13306.8	22026.9	3462.8	2773.7	2657.6	2.44	3.84E-16	5.93E-15	PAS domain containing protein
19262906	MAA_08620	229.3	196.3	230.3	48.0	34.1	38.1	2.45	3.38E-64	3.43E-62	nmrA-like family protein
19255948	MAA_01662	77.5	64.2	68.4	11.4	9.0	16.1	2.53	4.95E-23	1.14E-21	GCN5-related N-acetyltransferase (GNAT) domain protein
19264901	MAA_10615	77.2	51.6	124.0	10.3	15.3	17.3	2.56	2.41E-10	2.09E-09	ScfA
19255934	MAA_01648	0.6	1.4	3.6	0.4	0.0	0.6	2.58	1.78E-02	4.06E-02	Metallopeptidase, catalytic domain protein
19259265	MAA_04979	864.5	876.4	1289.4	178.2	160.5	163.8	2.59	1.81E-72	2.16E-70	chloride channel protein
19262575	MAA_08289	46.5	55.0	20.3	5.1	4.2	10.6	2.62	6.93E-11	6.56E-10	cell surface protein (Mas1)
19263643	MAA_09357	420.9	300.0	319.5	73.1	47.3	48.8	2.62	1.23E-51	8.01E-50	hypothetical protein X797_010135
19262908	MAA_08622	5.4	4.0	6.6	1.3	0.4	0.9	2.65	4.67E-13	5.73E-12	polyketide synthase
19259065	MAA_04779	5.4	6.0	5.5	0.8	1.0	0.8	2.70	4.28E-14	5.66E-13	hypothetical protein MAA_04779
19258936	MAA_04650	811.0	899.3	667.2	133.9	114.6	95.8	2.79	5.60E-77	7.08E-75	hypothetical protein MAA_04650
19263642	MAA_09356	3571.1	2497.3	2417.6	549.2	325.8	338.3	2.81	1.96E-18	3.52E-17	hemeolysin E
19255954	MAA_01668	129.4	136.6	259.5	25.4	22.2	21.8	2.92	2.68E-16	4.18E-15	Phosphoesterase
19262891	MAA_08605	15.7	12.7	12.3	1.0	3.5	0.2	3.11	5.97E-07	3.41E-06	hypothetical protein MAA_08605
19256297	MAA_02011	5.8	11.4	28.4	2.1	2.2	0.9	3.14	5.95E-06	2.93E-05	hypothetical protein MAA_02011
19255945	MAA_01659	8.3	11.5	7.4	0.9	0.6	1.5	3.21	2.25E-14	3.06E-13	hypothetical protein MAA_01659
19262030	MAA_07744	20.0	20.5	20.8	1.7	1.0	2.8	3.47	1.54E-28	4.60E-27	glucose-methanol-choline oxidoreductase
19264911	MAA_10625	2.6	5.7	6.1	0.0	0.0	0.8	4.15	5.16E-03	1.38E-02	Major facilitator superfamily domain, general substrate transporter
19264950	MAA_10666	2.4	1.7	1.5	0.0	0.0	0.0	7.48	1.60E-02	3.72E-02	hypothetical protein MAA_10666
19263641	MAA_09355	0.2	1.0	0.9	0.0	0.0	0.0	12.77	1.63E-02	3.78E-02	cyanamide hydratase

Table S4. A list of genes co-dysregulated in the Δ*brlA* and Δ*abaA*-1 mutants versus the WT strain of *M. roberstii*.

Gene_id	$\log_2$ Ratio*		Description
	Δ*brlA*/WT	Δ*abaA*/WT	
MAA_09352	-17.89	-5.08	hypothetical protein MAA_09352
MAA_09349	-16.54	-3.37	hypothetical protein X797_010127
MAA_08611	-15.53	-15.53	hypothetical protein X797_008967
MAA_01624	-14.04	-4.96	LysR family regulatory protein
MAA_01625	-13.34	-13.34	Thioesterase
MAA_08308	-9.84	-9.18	Peptidase aspartic, active site protein
MAA_09351	-9.23	-10.73	hypothetical protein MAA_09351
MAA_08339	-9.06	-11.15	hypothetical protein MAA_08339
MAA_04865	-7.91	-8.85	Peptidoglycan-binding lysin domain protein
MAA_08347	-7.30	-4.10	phytanoyl-CoA dioxygenase
MAA_01653	-7.10	-1.74	hypothetical protein MAA_01653
MAA_08370	-6.64	-2.82	polyketide synthase
MAA_04645	-6.20	-5.14	GPR1/FUN34/yaaH family protein
MAA_10665	-5.98	-5.16	hypothetical protein X797_001079
MAA_04450	-5.54	-5.37	Glycoside hydrolase, superfamily
MAA_09350	-5.52	-2.83	hypothetical protein MAA_09350
MAA_10602	-5.14	-5.55	subtilisin-like protease
MAA_08610	-5.05	-1.34	Protein kinase domain containing protein
MAA_08341	-4.86	-3.84	hypothetical protein MAA_08341
MAA_02359	-4.56	-3.25	Carbohydrate-binding WSC
MAA_10600	-4.50	-6.30	proteinrelated to zinc finger protein odd-paired-like (opl)
MAA_02183	-4.42	-1.11	Glucose-repressible protein Grg1
MAA_05017	-4.39	-1.45	Extracellular membrane protein, CFEM domain protein
MAA_08289	-4.05	2.62	cell surface protein (Mas1)
MAA_01642	-3.63	-2.68	fatty acid synthase subunit alpha reductase
MAA_01639	-3.63	-2.31	peptide synthetase
MAA_02376	-3.57	-3.09	Aromatic-ring hydroxylase-like protein
MAA_07718	-3.45	-3.04	hypothetical protein MAA_07718
MAA_08286	-3.37	-2.11	dehydrogenase/reductase SDR family
MAA_07742	-3.30	-5.36	subtilisin-like protease
MAA_08613	-3.30	-3.53	ntf2-like domain protein
MAA_01883	-3.29	-2.47	ABC-2 type transporter
MAA_05026	-3.22	-3.13	galactosyltransferase superfamily protein
MAA_01901	-3.20	-2.83	DUF967 domain protein
MAA_04690	-3.10	-2.57	DUF1479 domain protein
MAA_01637	-3.07	-3.20	hypothetical protein MAA_01637
MAA_08290	-3.02	1.99	hypothetical protein MAA_08290
MAA_10702	-2.88	-2.66	D-xylulose reductase A
MAA_10599	-2.85	1.55	leucine rich repeat domain containing protein
MAA_01638	-2.84	-1.57	canalicular multispecific organic anion transporter 1
MAA_10641	-2.71	-1.79	carbohydrate esterase family 1 protein
MAA_10709	-2.63	-2.68	nicotinamide N-methyltransferase
MAA_02039	-2.59	-2.58	Glutathione S-transferase/chloride channel
MAA_07741	-2.53	-3.83	Formate/nitrite transporter
MAA_02452	-2.37	-3.33	alcohol dehydrogenase
MAA_01629	-2.20	-1.30	GCN5-related N-acetyltransferase (GNAT) domain protein
MAA_09390	-2.19	-2.46	putative protein family Cys-rich
MAA_04981	-2.16	-1.40	Extracellular membrane protein, 8-cysteine region, CFEM
MAA_04909	-2.15	-1.17	major facilitator superfamily transporter
MAA_01641	-2.14	-1.32	ankyrin repeat protein
MAA_02090	-2.12	-1.51	GCN5-related N-acetyltransferase (GNAT) domain protein
MAA_08805	-2.12	-2.90	IDI-2 precursor
MAA_02027	-2.07	-1.69	hypothetical protein MAA_02027

MAA_04499	-2.05	-1.48	pyruvate decarboxylase
MAA_02413	-2.03	-2.53	C4-dicarboxylate transporter/malic acid transporter
MAA_02453	-2.03	-1.88	arrestin domain-containing protein
MAA_08327	-2.01	-1.66	hypothetical protein MAA_08327
MAA_04843	-1.97	-2.73	Basic-leucine zipper (bZIP) transcription factor
MAA_05008	-1.97	-1.04	taurine catabolism dioxygenase TauD
MAA_04650	-1.96	2.79	hypothetical protein MAA_04650
MAA_08570	-1.85	-1.20	glyoxalase family protein
MAA_04982	-1.82	-2.01	protein-arginine deiminase type-4
MAA_04710	-1.75	-1.14	Glutathione S-transferase/chloride channel
MAA_04917	-1.75	-1.15	Stress responsive alpha-beta barrel
MAA_04866	-1.73	-1.12	SAM-dependent methyltransferase
MAA_07644	-1.73	-1.10	hypothetical protein MAA_07644
MAA_02032	-1.69	-1.42	3-phytase
MAA_04537	-1.68	-2.56	Major facilitator superfamily domain protein
MAA_08621	-1.62	2.34	f420-dependent NADP reductase
MAA_02480	-1.60	-2.12	NAD-specific glutamate dehydrogenase
MAA_04750	-1.58	-1.83	
MAA_08623	-1.53	2.06	phytanoyl-CoA dioxygenase
MAA_10691	-1.53	-1.23	S-adenosyl-L-methionine-dependent methyltransferase
MAA_07740	-1.48	-1.10	muconate cycloisomerase I, MLE
MAA_01997	-1.46	-1.30	Carboxylesterase type B, active site protein
MAA_02377	-1.45	-1.62	ATP-binding cassette transporter ifT1
MAA_04961	-1.45	1.26	vacuolar triacylglycerol lipase
MAA_09347	-1.40	-1.28	transmembrane amino acid transporter
MAA_10670	-1.35	1.00	hypothetical protein MAA_10670
MAA_10681	-1.34	1.11	hypothetical protein MAA_10681
MAA_10682	-1.34	-1.27	WD40 domain protein
MAA_01953	-1.32	-1.05	dipeptidyl aminopeptidase/acylaminoacyl peptidase
MAA_09396	-1.30	-1.06	Alcohol dehydrogenase superfamily, zinc-type
MAA_01935	-1.29	-1.19	integral membrane protein
MAA_04788	-1.28	-1.31	dopa 4,5-dioxygenase
MAA_08806	-1.27	-3.44	Aminoglycoside phosphotransferase
MAA_09381	-1.27	-1.55	protoporphyrinogen oxidase
MAA_01702	-1.19	1.12	Chloramphenicol acetyltransferase-like domain protein
MAA_04685	-1.06	-1.13	hypothetical protein MAA_04685
MAA_02232	1.05	1.21	hypothetical protein MAA_02232
MAA_10686	1.09	1.33	WD repeat containing protein 44
MAA_04608	1.10	1.41	proteinrelated to alpha-glucosidase b
MAA_04821	1.13	1.23	hypothetical protein MAA_04821
MAA_08306	1.13	-2.03	Ankyrin repeat protein
MAA_07744	1.23	3.47	glucose-methanol-choline oxidoreductase
MAA_02251	1.34	1.94	chromosomal organization and DNA repair protein Mms21
MAA_08834	1.36	1.11	multidrug resistance-associated protein 1
MAA_01715	1.61	1.29	DOMON domain protein
MAA_08605	2.08	3.11	hypothetical protein MAA_08605
MAA_02250	2.28	2.44	PAS domain containing protein
MAA_02042	2.29	1.09	3-oxo-5-alpha-steroid 4-dehydrogenase
MAA_02321	2.34	2.41	Major facilitator superfamily domain, general substrate transporter
MAA_09359	2.54	1.76	Pfs, NACHT and WD domain protein
MAA_04780	2.60	1.33	hypothetical protein MAA_04780
MAA_04779	3.37	2.70	hypothetical protein MAA_04779
MAA_09358	3.45	2.05	hypothetical protein MAA_09358
MAA_09357	4.20	2.62	hypothetical protein X797_010135
MAA_09356	4.21	2.81	hemeolysin E

* Table entries marked in red denote those genes codysregulated at opposite directions.

Table S5. Counts of DEGs enriched to GO terms of three categories in the Δ*brlA* mutant of *M. robertsii*.

GO_ID	GO_Term	GO_Category	*p*-value	Count of DEGs	
				Up	Down
GO:0008150	biological_process	Biological Process	4.62E-02	16	52
GO:0055114	obsolete oxidation-reduction process	Biological Process	1.49E-02	4	10
GO:0035690	cellular response to xenobiotic stimulus	Biological Process	3.32E-02	0	5
GO:1900557	emericellamide biosynthetic process	Biological Process	1.16E-02	0	3
GO:1900560	austinol biosynthetic process	Biological Process	1.16E-02	1	2
GO:1900563	dehydroaustinol biosynthetic process	Biological Process	1.16E-02	1	2
GO:0030163	protein catabolic process	Biological Process	3.45E-02	0	2
GO:0033194	response to hydroperoxide	Biological Process	1.24E-02	1	1
GO:0052699	ergothioneine biosynthetic process	Biological Process	1.24E-02	1	1
GO:0052707	N-alpha,N-alpha,N-alpha-trimethyl-L-histidine biosynthesis from histidine	Biological Process	1.24E-02	1	1
GO:1903253	hercynylcysteine sulfoxide biosynthetic process	Biological Process	1.24E-02	1	1
GO:1903255	hercynylselenocysteine biosynthetic process	Biological Process	1.24E-02	1	1
GO:1903257	selenoneine biosynthetic process	Biological Process	1.24E-02	1	1
GO:0006808	regulation of nitrogen utilization	Biological Process	1.24E-02	1	1
GO:1902181	verruculogen biosynthetic process	Biological Process	3.45E-02	1	1
GO:0005575	cellular_component	Cellular Component	1.45E-05	23	94
GO:0016021	integral component of membrane	Cellular Component	2.85E-03	3	11
GO:0005886	plasma membrane	Cellular Component	2.45E-02	0	7
GO:0003674	molecular_function	Molecular Function	4.41E-03	17	57
GO:0000166	nucleotide binding	Molecular Function	5.48E-03	2	4
GO:0016887	ATP hydrolysis activity	Molecular Function	4.79E-02	0	4
GO:0008559	ABC-type xenobiotic transporter activity	Molecular Function	1.37E-03	0	3
GO:0017111	nucleoside-triphosphatase activity	Molecular Function	1.89E-03	1	3
GO:0005506	iron ion binding	Molecular Function	4.19E-02	2	3
GO:0052716	hydroquinone:oxygen oxidoreductase activity	Molecular Function	1.24E-02	0	2
GO:0015179	L-amino acid transmembrane transporter activity	Molecular Function	1.24E-02	0	2
GO:0015297	antiporter activity	Molecular Function	1.24E-02	0	2
GO:0016757	glycosyltransferase activity	Molecular Function	1.24E-02	0	2
GO:0052689	carboxylic ester hydrolase activity	Molecular Function	3.41E-02	1	2
GO:0016705	oxidoreductase activity, acting on paired donors, with incorporation or reduction of molecular oxygen	Molecular Function	3.62E-02	2	2
GO:0044876	hercynylselenocysteine synthase	Molecular Function	1.24E-02	1	1
GO:0052706	histidine N-methyltransferase activity	Molecular Function	1.24E-02	1	1
GO:0061686	hercynylcysteine sulfoxide synthase activity	Molecular Function	1.24E-02	1	1
GO:0016614	oxidoreductase activity, acting on CH-OH group of donors	Molecular Function	3.45E-02	1	1

Table S6. Counts of DEGs enriched to GO terms of three categories in the Δ*abaA*-1 mutant of *M. robertsii*.

GO_ID	GO_Term	GO_Category	*p*-value	Count of DEGs	
				Up	Down
GO:1900557	emericellamide biosynthetic process	Biological Process	5.99E-03	2	1
GO:0009405	obsolete pathogenesis	Biological Process	9.94E-03	3	3
GO:0031410	cytoplasmic vesicle	Cellular Component	7.82E-03	1	1
GO:0005575	cellular_component	Cellular Component	1.09E-02	39	46
GO:0016021	integral component of membrane	Cellular Component	2.50E-02	3	7
GO:0003674	molecular_function	Molecular Function	1.21E-03	23	40
GO:0016747	acyltransferase activity, transferring groups other than amino-acyl groups	Molecular Function	8.18E-03	2	2
GO:0052689	carboxylic ester hydrolase activity	Molecular Function	1.83E-02	2	1
GO:0004668	protein-arginine deiminase activity	Molecular Function	2.21E-02	1	1
GO:0004364	glutathione transferase activity	Molecular Function	4.16E-02	0	2
